# Mitochondrial reprogramming in cervical cancer: crosstalk with tumor immunity, HPV oncogenic signaling, and therapeutic resistance

**DOI:** 10.3389/fimmu.2026.1884498

**Published:** 2026-07-08

**Authors:** Li Pan, Peng Zhang, Dan Zhang, Gang Wang

**Affiliations:** Traditional Chinese Medicine Department, Zigong First People’s Hospital, Zigong, Sichuan, China

**Keywords:** cervical cancer, HPV, mitochondria, therapeutic resistance, tumor immunity

## Abstract

Cervical cancer remains a major malignancy in women worldwide despite advances in human papillomavirus (HPV) vaccination, screening, and multimodal treatment. Persistent high-risk HPV infection is the principal driver of cervical carcinogenesis, yet viral oncogenesis alone cannot fully explain tumor progression, immune escape, and therapeutic failure. Increasing evidence suggests that mitochondrial reprogramming is a critical adaptive process that links HPV-driven transformation to metabolic plasticity, tumor immunity, and resistance to therapy. Beyond their canonical role in ATP production, mitochondria regulate redox homeostasis, mitochondrial dynamics, mitophagy, apoptotic priming, and mitochondria-derived danger signaling, thereby shaping both tumor-cell fitness and the surrounding immune microenvironment. In cervical cancer, HPV-associated oncogenic signaling promotes metabolic and mitochondrial remodeling, while downstream mitochondrial processes help sustain malignant growth, buffer oxidative and therapeutic stress, and influence immune responsiveness. Emerging studies further indicate that mitochondrial stress signals, particularly mitochondrial DNA-mediated innate immune activation, may connect tumor metabolism with anti-tumor immunity and immunotherapeutic sensitivity. At the same time, mitochondrial respiration, redox adaptation, and quality-control mechanisms contribute to chemoresistance and broader treatment tolerance. In this review, we summarize current evidence on how HPV oncogenic signaling reshapes mitochondrial biology in cervical cancer and discuss how mitochondrial reprogramming influences tumor immunity, immune evasion, and therapeutic resistance. We also highlight emerging mitochondria-targeted strategies and propose future directions for mechanistic and translational research. Together, these insights position mitochondrial reprogramming as both a conceptual framework and a potential therapeutic vulnerability in cervical cancer.

## Highlights

- HPV oncogenic signaling promotes mitochondrial reprogramming that supports cervical cancer progression and stress adaptation.
- Mitochondrial dynamics, mitophagy, redox buffering, and mtDNA signaling connect tumor metabolism with immune regulation and immune evasion.
- Targeting mitochondrial vulnerabilities may improve therapeutic sensitivity and provide new combination strategies for cervical cancer.

## 1 Introduction

Cervical cancer remains one of the most common malignancies affecting women worldwide and continues to impose a substantial burden on public health, particularly in low- and middle-income countries (1–3). Although the implementation of prophylactic human papillomavirus (HPV) vaccination, organized screening programs, and improvements in standard treatments has significantly reduced incidence and mortality in some regions, cervical cancer remains a major cause of cancer-related death among women (4, 5). Importantly, a considerable proportion of patients are still diagnosed at a locally advanced stage, while others eventually develop recurrent, metastatic, or treatment-refractory disease. For these patients, long-term survival remains unsatisfactory despite multimodal treatment strategies. Current therapeutic approaches for cervical cancer mainly include surgery, radiotherapy, platinum-based chemotherapy, concurrent chemoradiotherapy, anti-angiogenic therapy, and, more recently, immunotherapy (6–9). However, the clinical efficacy of these interventions is frequently limited by intrinsic or acquired resistance, tumor heterogeneity, and the establishment of an immunosuppressive tumor microenvironment. In particular, although immune checkpoint blockade has introduced new opportunities for a subset of patients with recurrent or metastatic cervical cancer, the overall response rate remains modest, and durable benefit is achieved in only a fraction of cases. These limitations indicate that cervical cancer progression cannot be fully understood through HPV infection and canonical oncogenic pathways alone, but rather reflects a broader process of tumor adaptation involving metabolic rewiring, stress tolerance, and microenvironmental remodeling.

Persistent high-risk HPV infection is the initiating event in most cervical cancers, yet viral infection alone is insufficient to explain the full spectrum of malignant progression, immune escape, and therapeutic failure. Increasing evidence suggests that cervical cancer evolves through dynamic interactions between viral oncogenic signaling, host cell metabolic adaptation, and the surrounding stromal and immune contexture (10, 11). In this regard, metabolic plasticity has emerged as a key determinant of cancer cell survival under persistent oncogenic, oxidative, and therapeutic stress. Among the various organelles involved in this adaptive process, mitochondria are now recognized as central regulators of tumor cell fitness and microenvironmental communication.

Mitochondria have long been viewed primarily as intracellular powerhouses responsible for ATP generation through oxidative phosphorylation(OXPHOS). However, this classical view has been substantially expanded in recent years. In cancer, mitochondria are now understood as multifunctional signaling hubs that regulate not only energy production and biosynthetic metabolism, but also redox balance, apoptotic susceptibility, innate immune signaling, and cellular adaptation to stress (12–15). Their contributions to tumor biology extend far beyond bioenergetics and encompass multiple processes that directly influence cancer initiation, progression, and response to therapy. At the metabolic level, mitochondria integrate the utilization of glucose, amino acids, and lipids to support anabolic growth and survival. At the same time, they generate reactive oxygen species (ROS), which can function as signaling molecules at moderate levels but become cytotoxic when accumulated excessively. Mitochondria also control the release of pro-apoptotic factors, thereby influencing the threshold for cell death under genotoxic or therapeutic stress (16–18). In addition, mitochondrial dynamics, including continuous cycles of fusion and fission, are essential for maintaining organelle integrity, metabolic flexibility, and adaptation to environmental challenges. These processes are further coordinated by mitophagy, a specialized form of selective autophagy that removes damaged mitochondria and preserves mitochondrial quality control. Beyond these canonical functions, mitochondria also participate in immunologically relevant signaling. Mitochondrial DNA (mtDNA), mitochondrial ROS, and other mitochondria-derived danger signals can activate innate immune pathways, including cGAS-STING-related signaling, thereby linking intracellular stress to inflammatory and immune responses (19–21). This role is particularly important in cancer, where mitochondrial dysfunction may shape both tumor cell behavior and the immune landscape of the tumor microenvironment. Thus, mitochondrial abnormalities should no longer be regarded as passive byproducts of malignant transformation, but rather as active components of adaptive tumor reprogramming. In cervical cancer, mounting evidence indicates that mitochondrial network remodeling, respiratory adaptation, redox regulation, and mitochondrial quality control are closely associated with aggressive behavior and therapeutic response (22–24). Changes in mitochondrial function have been linked to enhanced proliferation, survival under treatment stress, altered apoptotic sensitivity, and resistance to platinum-based chemotherapy. These findings suggest that mitochondrial reprogramming is not a secondary consequence of cervical carcinogenesis, but a biologically meaningful process that contributes to disease evolution and clinical outcome.

The intersection of HPV oncogenic signaling, mitochondrial reprogramming, and tumor immunity represents a particularly important yet insufficiently integrated area in cervical cancer research. Most mechanistic studies have traditionally focused on HPV-driven disruption of cell-cycle control, especially through the actions of the viral oncoproteins E6 and E7 on p53 and retinoblastoma protein (25–27). While these pathways remain fundamental to cervical carcinogenesis, they do not fully capture the broader adaptive networks through which HPV-transformed cells sustain malignant growth, evade host immunity, and develop resistance to therapy. Emerging evidence indicates that HPV oncogenes influence cancer cell biology not only by deregulating proliferation and survival pathways, but also by remodeling cellular metabolism (28–30). HPV-associated signaling has been linked to enhanced glycolysis, altered mitochondrial metabolism, redox adaptation, and changes in mitochondrial homeostasis (31, 32). In other words, HPV does not merely initiate transformation; it may also help establish a metabolic and mitochondrial context that favors persistent growth under hostile conditions. This perspective is particularly relevant in cervical cancer, where tumor cells must adapt to chronic inflammatory signaling, oxidative stress, fluctuating nutrient availability, and repeated exposure to radiotherapy or chemotherapy. At the same time, mitochondrial reprogramming can influence tumor immunity through several non-mutually exclusive mechanisms. Mitochondria-derived ROS, metabolites, and mtDNA can function as signaling mediators that reshape innate immune sensing, inflammatory tone, and the composition of the tumor microenvironment (33, 34). These signals may either promote anti-tumor immune activation or, under persistent and dysregulated conditions, contribute to immune evasion and immunosuppressive remodeling. Moreover, mitochondrial fitness is likely to affect how cervical cancer cells respond to immune-mediated stress as well as cytotoxic treatments, thereby creating a mechanistic bridge between metabolic adaptation, immune escape, and therapeutic resistance. This framework provides the rationale for discussing mitochondrial reprogramming as a bridge between HPV-driven transformation, immune remodeling, and treatment response in cervical cancer.

This review discusses how HPV oncogenic signaling reshapes mitochondrial biology in cervical cancer and how mitochondrial reprogramming influences tumor immunity, immune evasion, and therapeutic resistance. We first summarize the upstream role of persistent HPV infection and viral oncogene activity in establishing a metabolically adaptive and mitochondria-centered malignant state. We then review the major features of mitochondrial reprogramming in cervical cancer, including alterations in bioenergetics, redox homeostasis, mitochondrial dynamics, mitophagy, and mitochondria-derived stress signaling. Next, we focus on the crosstalk between mitochondria and the tumor immune microenvironment, with particular emphasis on how mitochondrial signals may connect intracellular metabolic stress with innate immune activation, immune suppression, and variable responses to immunotherapy. We also discuss the role of mitochondrial adaptation in resistance to chemotherapy, radiotherapy, immune checkpoint blockade, and current immunotherapy-based combinations, with particular attention to how mitochondrial reprogramming may influence response to anti-PD-1/PD-L1 therapy in cervical cancer. Finally, we summarize emerging mitochondria-targeted strategies and highlight key knowledge gaps that must be addressed to translate mitochondria-based mechanisms into clinically meaningful biomarkers and therapeutic approaches for cervical cancer.

This narrative review was prepared through a structured literature search of PubMed, Web of Science, Scopus, and Google Scholar. The search primarily covered articles published up to 2026 and used combinations of the following terms: “cervical cancer,” “human papillomavirus,” “HPV,” “mitochondria,” “mitochondrial reprogramming,” “mitochondrial metabolism,” “oxidative phosphorylation,” “reactive oxygen species,” “mitophagy,” “mitochondrial dynamics,” “mtDNA,” “cGAS-STING,” “tumor immunity,” “immune evasion,” “immunotherapy,” “cisplatin resistance,” “radio resistance,” and “therapeutic resistance.” Relevant original studies, clinical studies, mechanistic investigations, and recent reviews were considered. Priority was given to studies directly related to cervical cancer or HPV-associated malignancies. When cervical cancer-specific evidence was limited, selected studies from broader cancer or immunometabolism research were included to support mechanistic interpretation, and such extrapolations were discussed cautiously. Articles were excluded if they were unrelated to mitochondrial biology, cervical cancer, HPV signaling, tumor immunity, or therapeutic resistance, or if they lacked sufficient mechanistic or translational relevance. The final literature selection was based on relevance to the conceptual framework of HPV-driven mitochondrial reprogramming, tumor-immune crosstalk, and treatment resistance in cervical cancer. In addition to summarizing published findings, this review critically evaluates the strength of available evidence, highlights unresolved controversies, and distinguishes well-supported mechanisms from preliminary or hypothesis-generating observations.

In this review, we distinguish between different levels of evidence whenever possible. Findings obtained directly from cervical cancer tissues, cervical cancer cell lines, patient-derived samples, or cervical cancer models are described as cervical cancer-specific evidence. Studies performed in HPV-associated malignancies are used to support HPV-related mechanistic interpretation. When direct cervical cancer data are limited, selected evidence from broader oncology, mitochondrial biology, or tumor immunology studies is discussed as mechanistic extrapolation rather than as definitive cervical cancer-specific evidence. This distinction is particularly important for topics such as mitochondrial regulation of tumor immunity, immunometabolic competition, cGAS-STING signaling, and mitochondria-targeted therapeutic strategies, where cervical cancer-specific evidence is emerging but remains incomplete.

## 2 HPV oncogenic signaling as an upstream driver of mitochondrial reprogramming

### 2.1 Persistent HPV infection as the initiating event in cervical carcinogenesis

Persistent infection with high-risk human papillomaviruses is the central etiological event in the development of cervical cancer. Among the oncogenic HPV genotypes, HPV16 and HPV18 account for the majority of cases and have been most extensively linked to malignant progression (35, 36). In most individuals, HPV infection is transient and cleared by host immunity. However, when viral persistence occurs, long-term expression of viral oncogenes progressively disrupts epithelial homeostasis, promotes genomic instability, and drives the transition from precancerous lesions to invasive carcinoma. This process is not only dependent on viral presence per se, but also on the sustained biological consequences of chronic viral-host interaction.

The transforming potential of high-risk HPV is primarily mediated by the viral oncoproteins E6 and E7. E6 promotes degradation of p53 and impairs p53-dependent apoptosis, DNA damage responses, and senescence, whereas E7 inactivates retinoblastoma family proteins, thereby releasing E2F transcription factors and promoting unscheduled cell-cycle progression (37–41). Together, these oncogenes uncouple proliferation from normal checkpoint control and create a permissive environment for malignant transformation. However, the impact of E6 and E7 extends well beyond canonical cell-cycle deregulation. Increasing evidence indicates that these viral proteins also reshape the metabolic architecture of infected cells, facilitating long-term survival and proliferation under conditions of persistent stress.

Importantly, HPV-driven carcinogenesis unfolds in a tissue environment characterized by chronic inflammation, repeated oxidative injury, and fluctuating nutrient and oxygen availability. Under these conditions, transformed cells must acquire adaptive mechanisms that allow them to maintain growth, tolerate stress, and evade elimination by host defenses. Mitochondrial reprogramming is increasingly recognized as one such adaptation. Rather than being a downstream epiphenomenon, mitochondrial remodeling may represent an early and functionally relevant consequence of HPV oncogene activity, helping transformed cervical epithelial cells accommodate both the energetic demands and the stress burden of malignant progression.

Thus, persistent HPV infection should be viewed not merely as the trigger for oncogenic signaling, but as the upstream biological force that establishes a permissive context for metabolic rewiring, mitochondrial adaptation, and tumor microenvironmental remodeling. This perspective broadens the understanding of HPV-mediated carcinogenesis from a predominantly nuclear and cell-cycle-centered model to one that includes organelle-level reprogramming and adaptive stress biology.

### 2.2 HPV oncogenes rewire glucose metabolism and mitochondrial function

A growing body of evidence suggests that HPV oncogenes actively reshape cellular metabolism, thereby contributing to the establishment of a malignant phenotype that is both proliferative and stress tolerant. One of the most consistent observations is the enhancement of glycolytic activity in HPV-positive cervical cancer cells. Viral oncogene expression has been associated with upregulation of key glycolytic enzymes, particularly hexokinase 2 (HK2), which catalyzes the first committed step of glucose metabolism and is widely recognized as a major driver of cancer-associated glycolytic flux (42, 43). The induction of HK2 provides cervical cancer cells with not only a bioenergetic advantage but also a metabolic framework that supports biosynthesis, redox buffering, and survival under adverse conditions.

However, the metabolic effects of HPV are unlikely to be restricted to glycolysis alone. Rather than enforcing a purely glycolytic state, HPV-associated signaling appears to promote a more flexible metabolic phenotype in which glycolytic enhancement is coordinated with mitochondrial adaptation (44–46). Such coordination is biologically plausible because highly proliferative cancer cells require not only rapid ATP production but also efficient integration of carbon substrates into anabolic and redox-supporting pathways. In this context, mitochondria remain indispensable even in tumors with elevated glycolysis. They provide intermediates for biosynthesis, regulate oxidative stress responses, and determine the threshold for stress-induced cell death. Therefore, HPV-mediated metabolic rewiring should be understood as a broader process that includes changes in mitochondrial function, rather than as a simple shift away from mitochondrial dependence.

Several signaling mediators may contribute to this reprogramming. HPV-associated oncogenic pathways intersect with regulators such as c-Myc, AMPK/LKB1-related networks, and stress-responsive metabolic programs, thereby creating a cellular state characterized by increased nutrient uptake, altered mitochondrial utilization, and enhanced survival capacity (47). In this setting, glycolysis and mitochondrial metabolism are not opposing programs but interconnected components of an adaptive metabolic system. By promoting this integrated rewiring, HPV oncogenes may help cervical cancer cells sustain malignant growth while also increasing their capacity to withstand oxidative, immune, and therapeutic stress.

This concept has important implications for how cervical cancer metabolism is interpreted. It suggests that HPV-driven metabolic alterations are not merely passive reflections of rapid growth, but active components of oncogenic programming that shape mitochondrial behavior and influence downstream phenotypes such as immune evasion and treatment response. As a result, metabolic and mitochondrial vulnerabilities downstream of HPV may represent more actionable therapeutic targets than viral infection alone, particularly in advanced disease.

### 2.3 HPV-associated regulation of mitochondrial quality control and structural homeostasis

In addition to influencing metabolic flux, HPV-associated oncogenic signaling may affect the structural and quality-control systems that maintain mitochondrial integrity. Mitochondria are highly dynamic organelles whose function depends on coordinated regulation of membrane architecture, mitochondrial DNA organization, proteostasis, and turnover of damaged organelles. In cancer cells, disruption of these homeostatic processes can either compromise survival or, if adaptively reprogrammed, confer significant selective advantages. Evidence from cervical cancer suggests that viral oncogenesis may intersect with these mitochondrial quality-control networks in ways that support malignant progression (48, 49). One important aspect of mitochondrial homeostasis involves the maintenance of mitochondrial membrane organization and nucleoid-associated protein complexes. Proteins such as ATPase family AAA domain-containing protein 3A (ATAD3A), which are implicated in mitochondrial dynamics, mtDNA maintenance, cholesterol trafficking, and mitochondrial structural integrity, have been reported to be dysregulated in cervical cancer and associated with HPV-related disease biology (50). This is notable because alterations in such mitochondrial scaffold and maintenance proteins may influence not only bioenergetic capacity, but also susceptibility to apoptosis, oxidative injury, and metabolic stress. In other words, viral transformation may create selective pressure for a mitochondrial architecture that is optimized for survival rather than for normal epithelial physiology.

Mitochondrial quality control also depends on the ability of cells to detect and remove dysfunctional organelles before they trigger catastrophic oxidative stress or apoptotic signaling. Although the full mechanisms remain incompletely defined in cervical cancer, it is increasingly plausible that HPV-transformed cells rely on altered mitochondrial surveillance and repair programs to maintain fitness in the face of chronic oncogenic and inflammatory stress. Such changes may include reorganization of mitochondrial membranes, altered turnover of damaged proteins, and enhanced capacity to preserve mitochondrial function during repeated exposure to genotoxic therapies. From a conceptual standpoint, HPV-associated mitochondrial remodeling should therefore be viewed at two levels: functional and structural. Functionally, HPV signaling supports metabolic adaptation and stress resistance; structurally, it may promote a mitochondrial state that is more stable, more repairable, and more compatible with long-term malignant survival. These features likely reinforce one another. A cancer cell with an adaptable metabolic program but unstable mitochondrial architecture would remain vulnerable to stress, whereas one that combines metabolic plasticity with robust mitochondrial maintenance gains a much stronger survival advantage. This framework helps explain why mitochondrial quality control is likely to be a crucial but still underappreciated dimension of HPV-driven cervical carcinogenesis.

### 2.4 Conceptual summary: HPV signaling establishes the metabolic and mitochondrial context for malignant progression

**Figure 1:**
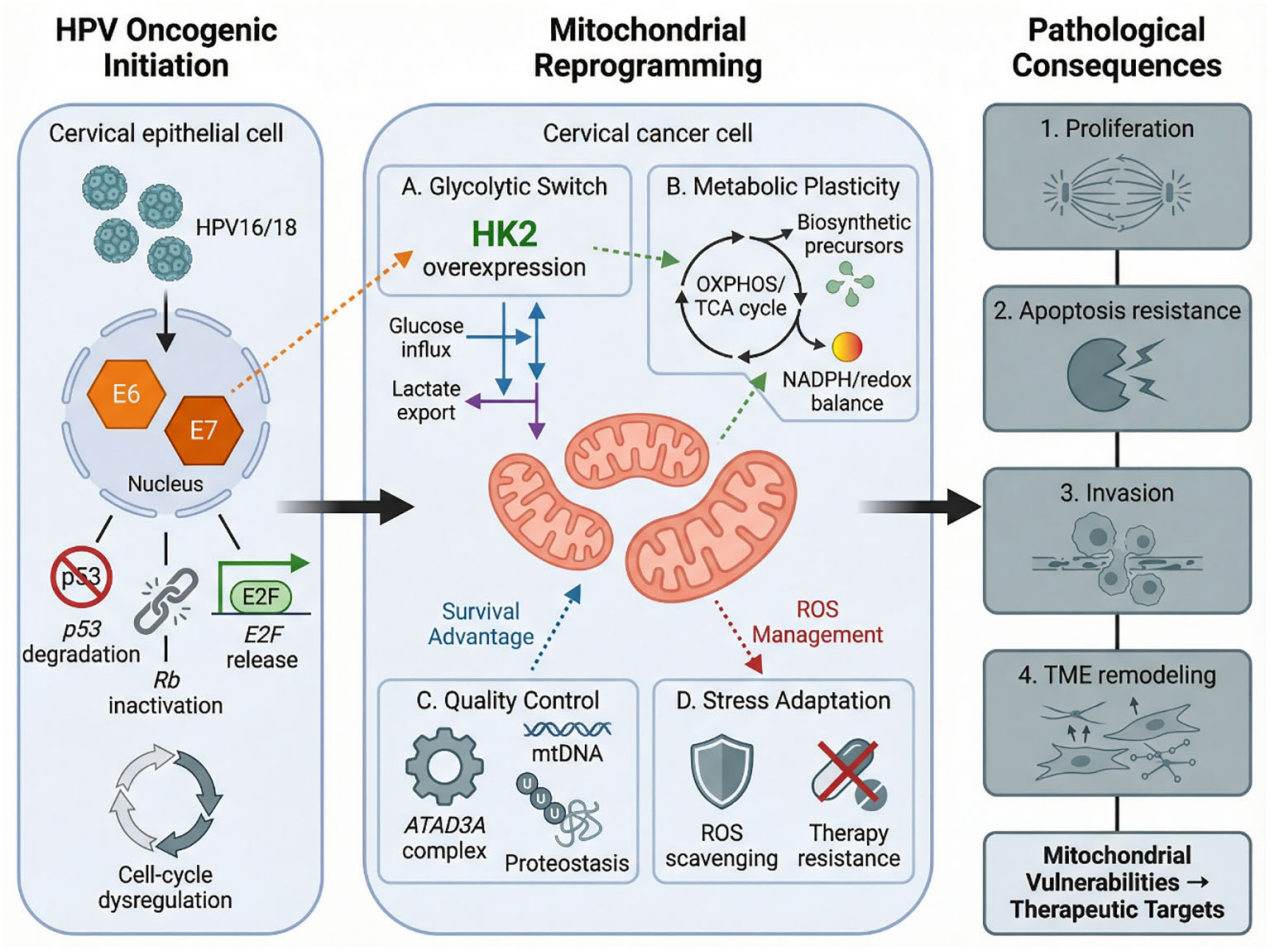
HPV oncogenic signaling drives mitochondrial reprogramming in cervical cancer. Persistent high-risk HPV infection, mainly through E6/E7 oncoproteins, promotes metabolic rewiring and mitochondrial adaptation in cervical cancer cells. These changes include HK2-associated glycolytic enhancement, flexible mitochondrial metabolism, mitochondrial quality control, structural homeostasis, and stress tolerance, collectively supporting malignant progression and creating potential mitochondrial therapeutic vulnerabilities.

Taken together, current evidence supports a model in which HPV oncogenic signaling contributes to mitochondrial reprogramming in cervical cancer. Beyond disrupting p53- and Rb-dependent cell-cycle control, HPV oncogenes appear to promote glycolytic activation, mitochondrial metabolic adaptation, redox buffering, and mitochondrial structural homeostasis. These changes may help transformed cervical epithelial cells survive chronic inflammation, oxidative stress, immune pressure, and therapeutic injury. Therefore, HPV-associated mitochondrial remodeling should be viewed as an adaptive extension of viral oncogenesis rather than as a secondary metabolic byproduct. This section provides the foundation for the following discussion of mitochondrial dynamics, mitophagy, redox control, mtDNA signaling, immune regulation, and therapeutic resistance. As shown in **Figure 1**, persistent high-risk HPV infection drives a mitochondria-centered adaptive program that integrates metabolic rewiring, mitochondrial quality control, and stress adaptation in cervical cancer.

## 3 Core features of mitochondrial reprogramming in cervical cancer cells

### 3.1 Mitochondrial bioenergetic remodeling

One of the central features of mitochondrial reprogramming in cervical cancer is the remodeling of cellular bioenergetics. Although enhanced aerobic glycolysis is a hallmark of many tumors, cancer cell metabolism is rarely a simple binary switch from oxidative phosphorylation to glycolysis (51, 52). Instead, cervical cancer cells appear to acquire a flexible bioenergetic phenotype in which glycolytic activation coexists with persistent mitochondrial function. This metabolic plasticity allows tumor cells to adapt to heterogeneous microenvironmental conditions, including fluctuations in oxygen tension, nutrient availability, oxidative burden, and therapy-induced stress.

Mitochondrial bioenergetic remodeling enables cervical cancer cells to maintain ATP production, preserve biosynthetic capacity, sustain redox balance, and retain respiratory complex function under demanding conditions (53, 54). Even when glycolysis is upregulated, mitochondrial respiration remains important for generating tricarboxylic acid cycle intermediates, supporting nucleotide and lipid synthesis, and maintaining mitochondrial membrane potential. Mechanistically, mitochondrial bioenergetic remodeling can support cervical cancer proliferation through several interconnected routes (55, 56). Sustained electron transport and mitochondrial membrane potential maintain ATP production and enable continued activity of biosynthetic pathways required for cell-cycle progression (57, 58). TCA cycle intermediates, such as citrate, α-ketoglutarate, and oxaloacetate-derived aspartate, provide carbon and nitrogen sources for lipid synthesis, nucleotide synthesis, amino acid metabolism, and epigenetic regulation (59, 60). At the same time, mitochondrial metabolism contributes to NADPH generation and glutathione-dependent antioxidant buffering, allowing proliferating cervical cancer cells to tolerate ROS produced by oncogenic signaling and rapid metabolic flux. Moderate mitochondrial ROS may further function as proliferative signaling molecules, whereas excessive ROS can trigger mitochondrial apoptosis or growth arrest (61). Therefore, mitochondrial bioenergetic remodeling promotes cervical cancer proliferation not simply by increasing ATP availability, but by coordinating energy production, biosynthetic precursor supply, redox balance, and survival signaling. Importantly, the preservation of mitochondrial respiration depends not only on substrate availability but also on accurate assembly and maturation of oxidative phosphorylation complexes (62). Complex I and complex III are major sites of electron transfer and ROS generation, whereas complex IV activity depends on coordinated assembly of mitochondrially encoded and nuclear-encoded subunits (63, 64). Recent pan-cancer analyses of mitochondrial complex assembly factors, including cytochrome c oxidase assembly factor 1 (COA1) and cytochrome c oxidase assembly protein 18 (COX18), suggest that mitochondrial protein assembly may influence tumor progression, mitochondrial signaling, and clinical outcome (65). Although these findings are not cervical cancer-specific, they support the broader concept that OXPHOS competence is shaped by respiratory complex assembly rather than by mitochondrial enzyme abundance alone. Therefore, in cervical cancer, the biological relevance of mitochondrial respiration should be interpreted not only in terms of ATP production but also in terms of complex assembly integrity, ROS control, TCA cycle support, and metabolic plasticity under HPV-driven and therapeutic stress. These functions are particularly relevant in rapidly proliferating or stress-exposed tumors, where biosynthetic and antioxidant demands are high. Therefore, mitochondrial metabolism in cervical cancer should not be viewed as residual or dispensable, but rather as an adaptive platform that complements glycolytic reprogramming. Recent studies further suggest that specific regulatory factors can enhance this bioenergetic adaptability by promoting oxidative metabolism or preserving mitochondrial respiratory function during treatment stress (66–70). Such findings support the concept that mitochondrial respiration contributes not only to basal tumor growth but also to the development of therapy tolerance. Cells capable of sustaining mitochondrial energy production and metabolic flexibility are more likely to survive under the selective pressures imposed by chemotherapy and radiotherapy.

Importantly, bioenergetic remodeling also has broader biological consequences beyond ATP generation. By altering electron transport activity and substrate utilization, cervical cancer cells can influence intracellular ROS levels, mitochondrial signaling outputs, and susceptibility to stress-induced apoptosis. Thus, mitochondrial bioenergetics represents a central organizing principle of cervical cancer adaptation, linking metabolic demand to survival, redox control, and downstream therapeutic response.

### 3.2 Mitochondrial dynamics: fusion, fission, and network remodeling

Mitochondria are highly dynamic organelles that continuously undergo fusion and fission in response to metabolic demand, stress conditions, and developmental cues. These dynamic processes are essential for maintaining mitochondrial integrity, distributing metabolic resources, removing damaged components, and matching mitochondrial architecture to cellular needs. In cancer, dysregulation of mitochondrial dynamics can confer major advantages by promoting proliferation, survival, invasion, and stress adaptation. Cervical cancer is no exception, and emerging evidence indicates that remodeling of the mitochondrial network is a meaningful component of tumor progression (71–73). Rather than existing as isolated static units, mitochondria form interconnected networks whose morphology reflects the balance between fusion and fission. A fused mitochondrial network may support efficient oxidative metabolism, resistance to transient stress, and redistribution of mitochondrial contents, whereas increased fission may facilitate rapid quality control, segregation of damaged organelles, altered cell-cycle progression, and adaptation to high proliferative demand. Tumor cells can exploit either direction of remodeling depending on context. What matters biologically is not the absolute degree of fusion or fission, but the ability to dynamically shift mitochondrial architecture to support malignant needs.

In cervical cancer, mitochondrial network remodeling has been linked to enhanced tumor growth and progression (74, 75). This suggests that oncogenic signaling pathways may actively reconfigure mitochondrial morphology in order to optimize both metabolic performance and survival signaling. Such reorganization may affect mitochondrial distribution within the cell, efficiency of energy transfer, and interaction with other organelles, including the endoplasmic reticulum. These structural changes can also influence ROS generation, apoptotic priming, ER–mitochondria communication, and sensitivity to chemotherapeutic stress. Mitochondrial dynamics should also be considered in relation to inter-organelle communication, particularly endoplasmic reticulum (ER)–mitochondria crosstalk (76, 77). Mitochondria-associated membranes (MAMs) provide specialized contact sites through which mitochondria and the ER exchange calcium, lipids, and stress signals. Changes in mitochondrial fusion, fission, and intracellular distribution may alter the extent and function of these contact sites, thereby influencing mitochondrial calcium uptake, lipid metabolism, ROS production, apoptotic priming, and ER stress responses (78). In cancer cells, ER–mitochondria communication may therefore contribute to metabolic adaptation and stress tolerance by coordinating mitochondrial bioenergetics with ER protein-folding capacity and unfolded protein response signaling (79). Although direct evidence in cervical cancer remains limited, this broader mechanism provides a useful framework for understanding how mitochondrial network remodeling may influence not only mitochondrial function but also ER homeostasis and tumor-cell survival.

At the same time, studies targeting fission-related pathways indicate that cervical cancer cells can be vulnerable to perturbation of mitochondrial dynamics. Excessive or forced mitochondrial fragmentation may impair proliferation or trigger cell-cycle arrest, whereas inappropriate preservation of mitochondrial network integrity may reduce adaptive capacity under stress. Together, these observations support the view that mitochondrial dynamics is not a passive reflection of tumor state, but an actively regulated determinant of cell fitness. In cervical cancer, the mitochondrial network appears to function as a flexible structural platform through which oncogenic signals are translated into bioenergetic adaptation, survival competence, and malignant plasticity.

### 3.3 Mitophagy and mitochondrial quality control as adaptive survival programs

Because mitochondria are continuously exposed to oxidative injury, metabolic strain, and genotoxic stress, cancer cells require efficient systems to monitor and maintain mitochondrial quality. Mitophagy, the selective autophagic removal of damaged or dysfunctional mitochondria, is a major component of this surveillance network. In cervical cancer, mitophagy is increasingly recognized as an adaptive survival mechanism that enables tumor cells to tolerate persistent oncogenic stress and therapy-induced mitochondrial damage (80–82). The biological logic of mitophagy in cancer is straightforward. Damaged mitochondria can become major sources of excessive ROS, loss of membrane potential, bioenergetic collapse, and release of pro-apoptotic or immunostimulatory molecules. If not removed in time, they may push the cell toward irreversible death. By selectively eliminating compromised mitochondria, mitophagy preserves a healthier mitochondrial pool, limits toxic stress accumulation, and maintains metabolic continuity. This protective function is especially relevant in cervical cancer cells exposed to cisplatin, radiation, or oxidative stress, all of which can induce substantial mitochondrial injury.

However, mitophagy in cancer is not simply a housekeeping process. It is better understood as a context-dependent stress adaptation program. Under therapeutic pressure, enhanced mitophagy can act as a buffering mechanism that allows tumor cells to survive otherwise lethal mitochondrial damage. In this setting, mitophagy contributes to treatment tolerance by lowering intracellular stress burden and preventing activation of mitochondrial apoptosis. This has major implications for cervical cancer, where resistance to platinum-based therapy remains a central clinical problem (83). Mitophagy also intersects with broader aspects of mitochondrial homeostasis, including dynamics, redox regulation, and metabolic plasticity. Fragmented mitochondria are often more readily targeted for removal, and selective elimination of dysfunctional organelles can reshape the overall bioenergetic state of the cell. Thus, mitophagy is closely integrated with mitochondrial remodeling rather than functioning as an isolated pathway. In cervical cancer, the balance between mitochondrial damage and mitochondrial clearance may be a decisive factor in determining whether cells undergo death or adapt to survive. This makes mitophagy a particularly important node linking mitochondrial stress to long-term malignant persistence.

### 3.4 Oxidative stress, redox buffering, and mitochondrial survival advantage

Mitochondria are both producers and targets of reactive oxygen species, placing them at the center of redox regulation in cancer cells. In cervical cancer, oxidative stress has a dual role. On the one hand, excessive ROS can damage mitochondrial membranes, proteins, and DNA, ultimately promoting apoptosis or other forms of cell death (84, 85). On the other hand, moderate or spatially controlled ROS production can function as a signaling mechanism that supports proliferation, adaptation, and tumor progression. The biological outcome therefore depends not simply on ROS abundance, but on the balance between ROS generation and the cell’s antioxidant buffering capacity.

Cancer cells often survive in a state of chronic oxidative pressure due to oncogenic signaling, mitochondrial dysfunction, inflammatory microenvironmental cues, and treatment-related injury. To persist under these conditions, they must reinforce redox homeostasis. This is achieved through coordinated upregulation of antioxidant systems, altered substrate utilization, and adaptive mitochondrial regulation. In cervical cancer, mitochondrial redox buffering likely contributes to both basal tumor fitness and therapy resistance by preventing ROS from crossing the threshold from signaling mediator to cytotoxic effector.

This redox adaptation may confer several selective advantages. First, it allows tumor cells to preserve mitochondrial function despite sustained oxidative stress. Second, it reduces activation of mitochondrial apoptosis and stress-induced cell death pathways. Third, it may support resistance to ferroptosis and other oxidative modes of therapy-induced lethality. These functions are especially relevant in the context of cisplatin treatment, which can increase intracellular and mitochondrial oxidative burden. Cells capable of rapidly restoring redox equilibrium are more likely to withstand treatment and persist. Importantly, redox regulation is tightly connected to metabolic state. Mitochondrial substrate flux, NADPH availability, glutathione metabolism, and electron transport chain activity all influence ROS production and detoxification (86, 87). Thus, oxidative stress in cervical cancer cannot be separated from the broader framework of mitochondrial reprogramming. Instead, redox buffering should be regarded as one of its functional outputs. The ability of cervical cancer cells to maintain a survivable oxidative state may represent a key determinant of malignant progression, immune interaction, and resistance to therapy.

### 3.5 mtDNA, mitochondrial stress signals, and danger signaling

Beyond metabolism and redox control, mitochondria also function as sources of intracellular danger signals. Among these, mitochondrial DNA has emerged as a particularly important mediator linking mitochondrial stress to inflammatory and immune signaling. Under conditions of mitochondrial dysfunction, membrane disruption, impaired mitophagy, or excessive oxidative injury, mtDNA may leak into the cytosol or extracellular space (88–90). Once mislocalized, mtDNA can be recognized as a damage-associated molecular pattern and activate innate immune sensing pathways. This phenomenon is especially relevant in cancer because it converts an intracellular organelle stress response into a broader signaling event with consequences for both tumor cells and the surrounding microenvironment. Cytosolic mtDNA can activate the cGAS-STING pathway, leading to downstream transcriptional programs associated with interferon signaling, inflammation, and altered immune responsiveness. In principle, such signaling may either enhance anti-tumor immunity or, if chronically dysregulated, contribute to maladaptive inflammatory states and immune escape. The context, magnitude, and duration of mitochondrial stress are therefore likely to determine whether mtDNA release is immunostimulatory or tumor supportive.

In cervical cancer, this axis is of particular interest because the disease arises in the setting of persistent viral oncogenesis and a highly relevant immune microenvironment. Mitochondrial stress signals may help explain how metabolic and organelle-level changes influence host immune recognition in a virus-associated tumor. Rather than being separate layers of biology, mitochondrial dysfunction and immune signaling may be mechanistically intertwined. This is especially important when considering therapeutic strategies that intentionally induce mitochondrial stress in order to stimulate immune activation. More broadly, mtDNA release exemplifies the expanding conceptual role of mitochondria in cancer. Mitochondria are not only metabolic engines or arbiters of apoptosis; they are also signaling organelles capable of shaping inflammatory tone and immune contexture. In cervical cancer, understanding how mitochondrial danger signals are generated, buffered, or exploited may prove essential for explaining both variable immune responsiveness and treatment outcomes.

### 3.6 Integrative view: mitochondrial reprogramming as a platform for cervical cancer cell fitness

**Figure 2:**
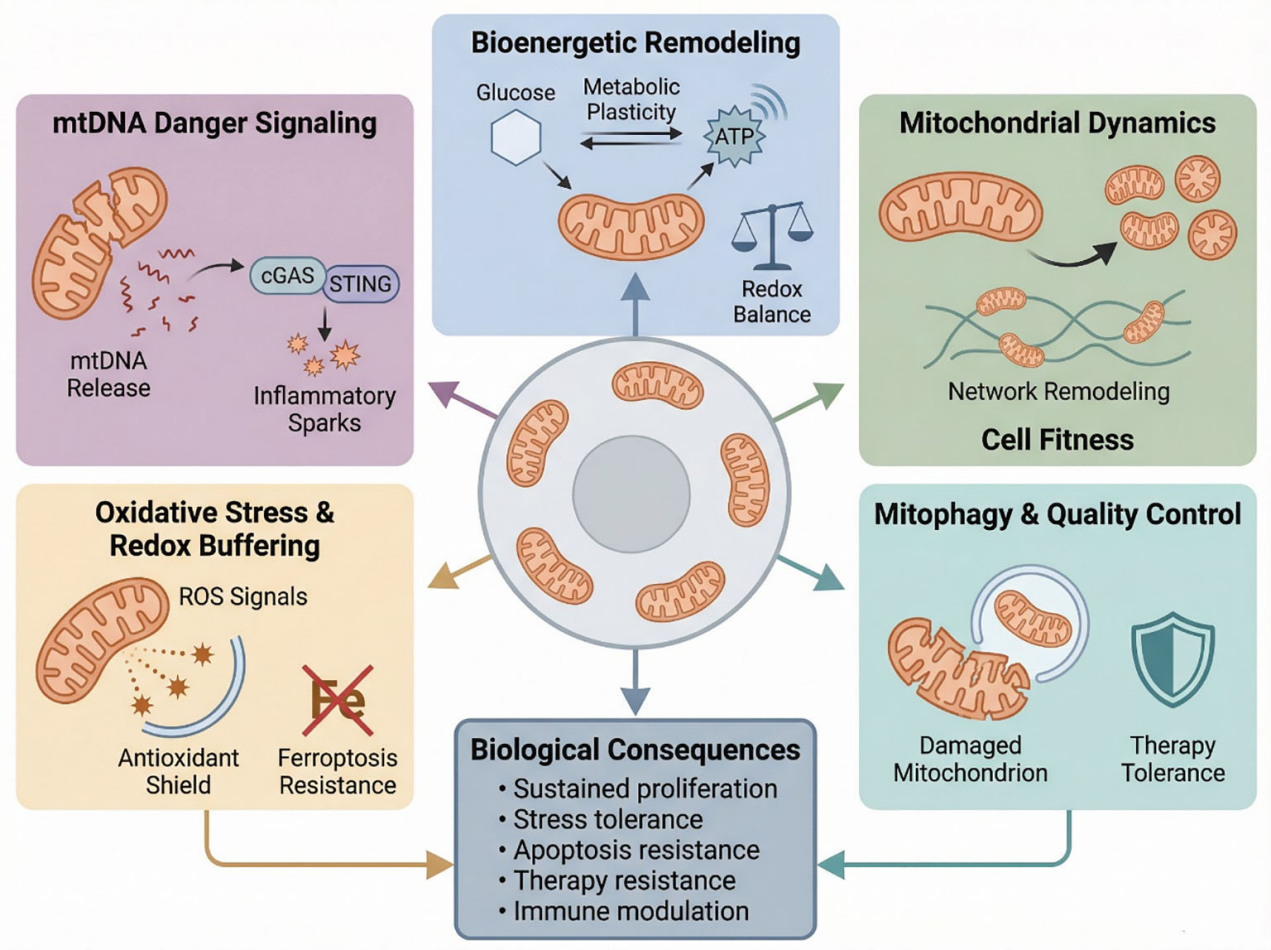
Core features of mitochondrial reprogramming in cervical cancer cells. Mitochondrial reprogramming in cervical cancer involves coordinated bioenergetic remodeling, dynamic network reorganization, adaptive mitophagy, reinforced redox buffering, and mtDNA-mediated danger signaling. Together, these interconnected processes support sustained proliferation, stress tolerance, resistance to apoptosis, therapeutic adaptation, and immune modulation.

The major features of mitochondrial reprogramming in cervical cancer include bioenergetic remodeling, mitochondrial network restructuring, mitophagy, redox buffering, and mtDNA-associated danger signaling. These processes are mechanistically interconnected: bioenergetic changes influence ROS production, ROS promotes mitochondrial damage, mitochondrial dynamics facilitates quality control, and persistent mitochondrial stress can generate immune-relevant signals. Together, these pathways form an adaptive mitochondrial fitness program that supports tumor-cell survival under metabolic, oxidative, and therapeutic stress. This framework also explains why mitochondrial biology is relevant to tumor immunity. Mitochondria regulate not only intracellular survival but also stress signals that can influence innate immune sensing, inflammatory tone, and immune-cell interactions. As illustrated in **Figure 2**, mitochondrial reprogramming provides a platform through which cervical cancer cells coordinate metabolic adaptation, stress tolerance, and immune modulation.

## 4 Mitochondria-tumor immunity crosstalk in cervical cancer

Before discussing mitochondria–immunity crosstalk, it should be emphasized that the strength of evidence varies across different mechanisms. Some observations, such as altered immune contexture in cervical cancer and emerging mtDNA-cGAS-STING-related findings, are supported by cervical cancer-specific studies (91, 92). In contrast, several concepts, including mitochondrial control of immune-cell fitness, immunometabolic competition, and mitochondrial stress-induced remodeling of the tumor immune microenvironment, are partly extrapolated from broader cancer immunology and mitochondrial biology research. Therefore, these mechanisms are discussed here as an evidence-graded framework rather than as uniformly established cervical cancer-specific conclusions.

### 4.1 Tumor immunity in cervical cancer: an HPV-shaped but immunosuppressed microenvironment

Cervical cancer occupies a unique position at the intersection of viral oncogenesis and tumor immunology. Because most cases arise from persistent high-risk HPV infection, cervical tumors are theoretically endowed with viral antigens that could serve as strong immunogenic targets. In principle, this should facilitate immune recognition and anti-tumor surveillance. However, clinical and biological observations reveal a more complex reality. Many cervical cancers progress despite the presence of viral antigens, and only a subset of patients derives durable benefit from immunotherapy. This paradox indicates that cervical cancer evolves within a microenvironment that is shaped by viral biology yet progressively remodeled toward immune suppression. This issue has become clinically important because immune checkpoint blockade is now integrated into the treatment landscape of cervical cancer. Anti-PD-1-based therapy is used in combination with chemotherapy, with or without bevacizumab, for selected patients with persistent, recurrent, or metastatic cervical cancer, and pembrolizumab combined with chemoradiotherapy has also entered the management of high-risk locally advanced disease. Therefore, mitochondrial regulation of tumor immunity should be interpreted not only as a biological mechanism but also as a potential determinant of response to current immunotherapy-based regimens.

The cervical tumor microenvironment contains a heterogeneous mixture of immune and stromal cells, including cytotoxic T lymphocytes, regulatory T cells, tumor-associated macrophages, dendritic cells, myeloid-derived suppressor cells, fibroblasts, endothelial cells, and metabolically active tumor cells (93). The composition and functional state of these populations are not static. Instead, they are continually influenced by chronic antigen exposure, inflammatory signals, hypoxia, metabolic competition, and therapy-induced stress (94, 95). Over time, these pressures can result in ineffective antigen presentation, T-cell dysfunction, increased suppressive myeloid activity, and a broader inflammatory milieu that favors tumor persistence rather than immune elimination. Importantly, immune suppression in cervical cancer is unlikely to be driven solely by classical checkpoint pathways. It also reflects the cumulative influence of metabolic and organelle-centered adaptive processes within tumor cells. Cancer cells that withstand immune attack often do so not only by altering ligand expression or cytokine production, but also by optimizing their metabolic state, redox homeostasis, and stress-response systems. In this context, mitochondria are increasingly relevant because they regulate multiple outputs that affect both intrinsic tumor survival and extrinsic immune signaling. Thus, the immune landscape of cervical cancer should be understood as both HPV-shaped and mitochondria-influenced. Persistent viral oncogenesis provides the initiating antigenic and inflammatory context, but mitochondrial reprogramming may help determine whether this context is translated into effective immune activation or into chronic, dysregulated, and ultimately tumor-supportive immune remodeling. This concept provides the foundation for examining mitochondria as active participants in tumor-immune crosstalk rather than as purely intracellular metabolic organelles.

### 4.2 Mitochondrial stress converts metabolic disturbance into immune signaling

One of the most important reasons mitochondria are relevant to tumor immunity is that they can convert intracellular stress into immunologically meaningful signals. As cancer cells undergo metabolic rewiring, redox imbalance, mitochondrial damage, and treatment-induced injury, mitochondria do not simply fail or adapt silently. Instead, they generate a range of outputs-including reactive oxygen species, oxidized metabolites, mitochondrial DNA, and altered membrane-derived signals-that can reshape inflammatory signaling and innate immune sensing. In this way, mitochondrial stress functions as a biological translator between tumor cell adaptation and immune system engagement (96–98). This principle is particularly relevant in cervical cancer, where tumor cells exist in a background of chronic viral oncogenic signaling and persistent immune pressure. HPV-driven transformation imposes sustained proliferative and metabolic demands, while the tumor microenvironment subjects cells to inflammatory stress, nutrient limitation, and intermittent therapeutic injury. Under these conditions, mitochondrial stress becomes almost inevitable. The critical question is not whether mitochondrial stress occurs, but how it is interpreted and managed by tumor cells and surrounding immune populations.

Moderate mitochondrial stress may enhance tumor cell plasticity and promote inflammatory programs that paradoxically support tumor progression. In contrast, more severe or poorly buffered stress may result in release of damage-associated signals capable of activating innate immunity. Thus, mitochondrial dysfunction exists on a spectrum, ranging from adaptive signaling that supports malignant fitness to danger signaling that can potentially expose tumor cells to immune attack. The boundary between these outcomes depends on the magnitude, duration, and cellular context of mitochondrial injury, as well as on the competence of downstream immune sensing pathways. This ability of mitochondria to bridge metabolic state and immune communication is especially important for understanding why tumor metabolism cannot be studied separately from tumor immunity. In cervical cancer, metabolic rewiring is not merely a cell-autonomous survival strategy; it may also influence how the tumor is perceived by immune cells, how inflammatory tone is established, and whether treatment-induced stress becomes immunogenic. Therefore, mitochondrial stress may represent a mechanistic interface linking organelle dysfunction to tumor immune ecology; however, in cervical cancer, this concept is currently supported by a combination of cervical cancer-specific observations and mechanistic extrapolation from broader tumor immunology studies.

### 4.3 mtDNA-cGAS-STING signaling as a central mitochondria-immunity axis

Among the mitochondria-derived immune signaling pathways, the mtDNA-cGAS-STING axis has emerging cervical cancer-specific support and is one of the most biologically relevant mechanisms linking mitochondrial stress to innate immune signaling in this disease. Nevertheless, whether mtDNA-cGAS-STING activation consistently predicts immunotherapy response or clinical outcome in cervical cancer remains insufficiently established and requires further validation in patient cohorts and immunocompetent models (91). Under conditions of mitochondrial damage or impaired quality control, mtDNA may escape from the mitochondrial matrix and accumulate in the cytosol. Because cytosolic DNA is normally restricted in healthy cells, this misplaced mtDNA is sensed as a danger signal by cyclic GMP-AMP synthase (cGAS), which in turn activates STING and downstream transcriptional programs involving type I interferon signaling and inflammatory mediators. This pathway links mitochondrial injury directly to innate immune activation.

In cervical cancer, this axis is particularly intriguing for several reasons. First, it provides a concrete mechanism by which mitochondrial reprogramming can influence anti-tumor immunity. Rather than affecting immune responses indirectly through general metabolic changes alone, damaged mitochondria may actively initiate immunostimulatory signaling through mtDNA release. Second, because cervical cancer is a virus-associated tumor, the cGA-STING pathway sits at a biologically meaningful intersection of antiviral sensing, tumor-intrinsic stress signaling, and microenvironmental immune regulation (99–101). Third, emerging evidence suggests that activation of this pathway can enhance anti-tumor immune responses in cervical cancer models, supporting its translational relevance. At the same time, the biological consequences of cGAS-STING activation are not necessarily uniform. Acute and appropriately triggered signaling may promote immune recognition and improve the efficacy of anti-tumor therapy, whereas chronic, dysregulated, or incomplete activation may generate inflammatory programs that are insufficient for tumor clearance or that become maladaptive over time. In addition, the functional impact of this pathway likely depends on the integrity of downstream signaling components, the extent of immune infiltration, and the broader immunometabolic context of the tumor microenvironment.

Nevertheless, the mtDNA-cGAS-STING axis offers one of the clearest examples of how mitochondria can act as immune-regulatory organelles in cervical cancer. It demonstrates that mitochondrial reprogramming is not limited to energy production or therapy resistance, but extends to direct modulation of innate immune signaling. This axis therefore provides a valuable mechanistic anchor for integrating mitochondrial biology with the immunological features of cervical cancer.

### 4.4 Mitochondrial reprogramming and immune evasion

Although mitochondria can generate immunostimulatory signals, mitochondrial reprogramming may also contribute to immune evasion. This apparent contradiction reflects the context-dependent nature of mitochondrial signaling in cancer. Tumor cells do not simply experience mitochondrial stress passively; they often remodel mitochondrial function in ways that minimize lethal damage, maintain metabolic flexibility, and reshape the surrounding environment to their advantage. As a result, mitochondrial adaptation may help convert potentially immunogenic stress into a state of chronic tolerance, suppressed immune activation, or dysfunctional inflammation.

Several mechanisms may underlie this process. First, improved mitochondrial quality control and redox buffering can reduce the accumulation of catastrophic mitochondrial damage, thereby limiting the release of danger signals that would otherwise enhance immune detection. Second, mitochondrial metabolic remodeling may alter the availability of metabolites that influence cytokine production, antigen presentation, or immune-cell activity in the microenvironment. Third, tumor cells with strong mitochondrial fitness are likely better equipped to withstand immune-mediated oxidative or apoptotic stress, allowing them to survive even in the presence of infiltrating effector cells. In cervical cancer, the direct mechanistic evidence linking mitochondrial adaptation to immune escape remains less extensive than in some other tumor types, but the conceptual basis is strong (102, 103). Accordingly, the role of mitochondrial adaptation in cervical cancer immune evasion should be interpreted as an emerging hypothesis supported by partial cervical cancer evidence and by stronger mechanistic evidence from other tumor contexts. The tumor microenvironment of cervical cancer frequently displays features of ineffective anti-tumor immunity despite viral antigenicity, suggesting that adaptive programs within tumor cells help blunt or redirect immune pressure. Mitochondrial reprogramming may be one such program, operating alongside checkpoint signaling, cytokine remodeling, and stromal interactions. By stabilizing tumor cell survival and modulating stress outputs, mitochondria may help create a state in which immune pressure persists but fails to eliminate the tumor. This view has important therapeutic implications. If mitochondrial adaptation contributes to immune escape, then targeting mitochondrial homeostasis may not only impair tumor cell survival directly but also restore or amplify anti-tumor immune recognition. In this sense, mitochondrial vulnerabilities may represent immunological as well as metabolic opportunities. Understanding how cervical cancer cells balance immunostimulatory mitochondrial stress with immune-evasive mitochondrial adaptation will therefore be critical for designing rational combination therapies.

### 4.5 Mitochondria and the immunometabolic competition within the tumor microenvironment

Tumor immunity is profoundly shaped by metabolic competition between cancer cells and infiltrating immune cells, and mitochondria are central to this contest. Within the cervical tumor microenvironment, both tumor cells and immune cells must operate under conditions of limited nutrients, fluctuating oxygen availability, high oxidative stress, and persistent inflammatory signaling. Because mitochondrial function is essential for bioenergetic adaptation and stress tolerance, the metabolic state of tumor cells can influence not only their own survival but also the functional capacity of surrounding immune populations. Highly adaptable tumor cells may consume and reallocate metabolic substrates in ways that create a hostile environment for effective immune responses. At the same time, mitochondrial dysfunction in immune cells themselves can impair cytotoxicity, cytokine production, memory formation, and sustained effector function. Although mitochondrial control of immunometabolic competition has been well described in broader tumor immunology, this issue has not yet been comprehensively dissected in cervical cancer. Therefore, its relevance to cervical cancer should currently be regarded as a plausible extrapolated framework rather than a fully established disease-specific mechanism. In a virus-associated malignancy where durable immune surveillance should, in theory, be possible, immunometabolic suppression may represent a major barrier to effective anti-tumor immunity.

Mitochondria likely influence this competition through several routes. Tumor-cell mitochondrial reprogramming can shape oxygen consumption, redox gradients, and the production of metabolites or signaling intermediates that influence immune-cell differentiation and function (104). Meanwhile, immune cells entering the tumor must maintain their own mitochondrial fitness in order to remain effective under chronic stimulation and metabolic scarcity. If tumor cells are metabolically optimized while immune cells become metabolically exhausted or dysfunctional, the result is a microenvironment that is immunologically populated yet functionally suppressed. This framework emphasizes that mitochondria should not be studied only within tumor cells. In future cervical cancer research, a more complete understanding will require attention to the mitochondrial states of both malignant and immune compartments. Such a perspective could help explain why tumors with apparent immune infiltration may still fail to respond to immunotherapy and why interventions that modify mitochondrial signaling could have system-wide effects on the tumor ecosystem.

### 4.6 Therapeutic activation of mitochondrial immune signaling to remodel the cervical cancer microenvironment

The ability of mitochondrial stress to engage immune pathways raises an important translational possibility: mitochondrial perturbation might be harnessed therapeutically to convert an immunosuppressive cervical tumor microenvironment into a more immunoreactive one (105–107). Rather than viewing mitochondrial dysfunction solely as a vulnerability that kills tumor cells directly, it may also be useful to consider whether controlled induction of mitochondrial stress can enhance immune recognition, stimulate innate sensing pathways, and improve response to immunotherapy or conventional treatment. This strategy is mechanistically attractive in cervical cancer because the tumor arises within a virologically and immunologically meaningful context; however, direct preclinical and clinical evidence supporting mitochondria-targeted immune remodeling in cervical cancer remains limited. If mitochondrial-targeted interventions can amplify mtDNA release, activate cGAS-STING signaling, or increase the immunogenic consequences of tumor-cell stress, they may help overcome the gap between antigenicity and effective immunity that characterizes many cervical cancers. Such approaches could, in principle, sensitize tumors to immune checkpoint blockade, radiotherapy, or chemotherapy by increasing inflammatory visibility and reducing the ability of tumor cells to buffer stress silently. At the same time, therapeutic activation of mitochondrial immune signaling must be approached with caution. Excessive or poorly controlled mitochondrial injury could damage beneficial immune cells, promote nonproductive inflammation, or select for tumor subclones with even stronger adaptive capacity. The success of such strategies will therefore depend on careful attention to timing, dosage, tumor context, and combination partners. Ideally, mitochondria-targeted immune activation would be paired with interventions that prevent tumor cells from neutralizing the resulting stress signals through mitophagy, redox buffering, or metabolic escape.

Overall, the mitochondria-immunity interface opens a promising therapeutic avenue in cervical cancer. It suggests that mitochondrial reprogramming is not only a hallmark of tumor adaptation but also a potential point of intervention for reshaping the immune microenvironment. By leveraging this interface, future therapies may be able to simultaneously weaken tumor fitness and enhance anti-tumor immunity. Thus, mitochondria-based immune activation should be considered a promising but still exploratory therapeutic concept in cervical cancer.

### 4.7 Integration with the current immunotherapy landscape in cervical cancer

The clinical relevance of mitochondria–immunity crosstalk should be considered in the context of the rapidly evolving immunotherapy landscape in cervical cancer (108, 109). Immune checkpoint inhibitors, particularly anti-PD-1/PD-L1-based strategies, have become important components of treatment for recurrent, metastatic, and locally advanced cervical cancer (110, 111). Pembrolizumab combined with platinum-based chemotherapy, with or without bevacizumab, has established immune checkpoint blockade as part of first-line therapy for selected patients with persistent, recurrent, or metastatic disease (112, 113). More recently, pembrolizumab combined with concurrent chemoradiotherapy has further extended the role of immunotherapy into high-risk locally advanced cervical cancer (114, 115). These clinical advances make it increasingly important to understand why some patients benefit from immunotherapy whereas others show primary resistance or incomplete response.

Mitochondrial reprogramming may influence immune checkpoint inhibitor response through several non-mutually exclusive mechanisms. First, tumor-cell mitochondrial metabolism can shape hypoxia, nutrient competition, and redox stress within the tumor microenvironment, thereby influencing T-cell infiltration and effector function. Tumors with high OXPHOS activity may consume oxygen and reinforce hypoxic or metabolically restrictive niches, potentially limiting cytotoxic T-cell activity and reducing responsiveness to PD-1/PD-L1 blockade. Second, mitochondrial quality control and mitophagy may reduce the accumulation of damaged mitochondria and limit mtDNA release, thereby weakening innate immune activation and type I interferon signaling (111, 116, 117). Conversely, excessive or therapy-induced mitochondrial stress may enhance mtDNA-cGAS-STING signaling and increase inflammatory visibility, which could improve sensitivity to immune checkpoint blockade when downstream immune pathways remain intact.

Third, mitochondrial redox adaptation may allow tumor cells to tolerate immune-mediated oxidative stress and resist cytotoxic lymphocyte-induced killing. In this context, mitochondrial fitness may function as a tumor-intrinsic resistance mechanism even when T cells are present (118–120). Fourth, the mitochondrial state of immune cells themselves is likely to influence immunotherapy efficacy. Functional CD8^+^ T cells, memory T cells, dendritic cells, and natural killer cells require adequate mitochondrial fitness to sustain cytokine production, antigen presentation, cytotoxicity, and long-term immune surveillance. Therefore, mitochondria-targeted combinations must be designed carefully so that they weaken tumor-cell mitochondrial adaptation without impairing anti-tumor immune-cell function.

These considerations are also relevant to current combination strategies. In chemoradiotherapy plus immunotherapy, radiation and platinum agents can induce mitochondrial damage, ROS accumulation, immunogenic stress, and potentially mtDNA-mediated innate immune activation. Mitochondrial reprogramming may determine whether this stress becomes immunostimulatory or is buffered by mitophagy and antioxidant programs. In chemotherapy, anti-angiogenic therapy, and immunotherapy combinations, mitochondrial metabolism may interact with hypoxia, vascular normalization, immune-cell trafficking, and myeloid suppression. Thus, mitochondrial biomarkers such as OXPHOS signatures, mitophagy markers, redox adaptation markers, mtDNA-cGAS-STING activation, and immune-cell metabolic fitness may help refine patient selection for immunotherapy-based combinations in cervical cancer.

### 4.8 Integrative view: mitochondria as immune-regulatory organelles in cervical cancer

**Figure 3:**
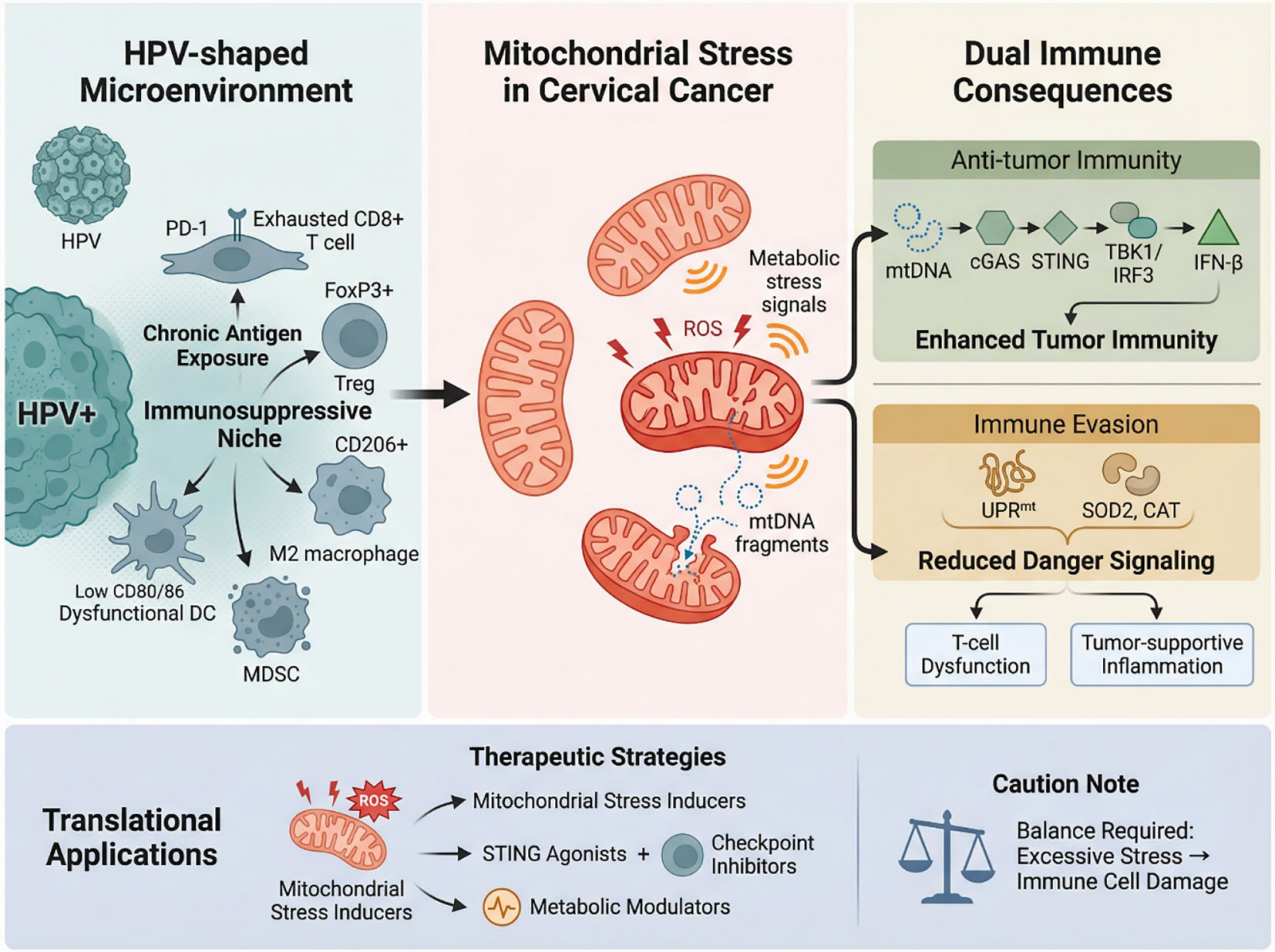
Mitochondria–tumor immunity crosstalk in cervical cancer. In cervical cancer, mitochondrial stress converts metabolic disturbance into immune-relevant signaling through ROS, mtDNA release, and cGAS–STING activation. These pathways may either enhance anti-tumor immunity or, when chronically buffered by mitochondrial adaptation, promote immune evasion and dysfunctional inflammation. Targeting this mitochondria–immunity interface may provide new opportunities to improve therapeutic response.

Taken together, available evidence supports an evidence-graded model in which mitochondria may function as immune-regulatory organelles in cervical cancer. In this model, mitochondrial metabolism, redox regulation, and mtDNA-cGAS-STING signaling have varying degrees of cervical cancer-specific support, whereas mitochondrial control of immune-cell fitness and immunometabolic competition remains more dependent on extrapolation from broader oncology literature. They influence tumor immunity not only through indirect effects on metabolism and survival, but also through active emission of stress signals, control of redox tone, and regulation of innate immune sensing pathways such as mtDNA-cGAS-STING. These functions position mitochondria at the interface between HPV-driven malignant adaptation and the broader immune behavior of the tumor microenvironment. This integrative view helps explain several otherwise disconnected observations in cervical cancer biology. It explains how a virus-associated tumor can remain immunologically visible yet clinically resistant; how metabolic adaptation can affect immune responsiveness; and how therapy-induced mitochondrial stress can sometimes result in immune activation rather than merely cytotoxicity. It also suggests that mitochondrial fitness is likely a determinant not only of tumor survival but of the quality of tumor-immune interaction. Most importantly, this framework creates a direct bridge to therapeutic resistance, the topic of the next section. If mitochondria regulate both stress adaptation and immune signaling, then mitochondrial reprogramming may be a key reason why cervical cancer cells survive chemotherapy, radiotherapy, and immune pressure simultaneously. Understanding this dual role is essential for identifying mitochondrial vulnerabilities that can be exploited in more effective treatment strategies. As shown in **Figure 3**, mitochondrial stress in cervical cancer acts as a key interface between tumor metabolism and immune regulation. Depending on the balance between danger signaling and adaptive buffering, mitochondrial reprogramming may either promote anti-tumor immune activation or contribute to immune evasion and dysfunctional inflammation.

## 5 Mitochondrial reprogramming as a driver of therapeutic resistance

### 5.1 Therapeutic resistance remains a central barrier in cervical cancer management

Despite substantial advances in prevention, diagnosis, and multimodal treatment, therapeutic resistance remains one of the most important challenges in cervical cancer management. For patients with locally advanced disease, concurrent chemoradiotherapy is a cornerstone of treatment, while recurrent or metastatic cases may receive platinum-based chemotherapy, anti-angiogenic agents, immune checkpoint inhibitors, or combinations of these modalities. However, many patients eventually experience incomplete response, disease relapse, or progressive resistance. This limitation is particularly evident in recurrent and metastatic cervical cancer, where durable control remains difficult to achieve and the survival benefit of newer therapies is still restricted to a subset of patients.

Traditionally, treatment failure in cervical cancer has been attributed to factors such as tumor heterogeneity, insufficient drug delivery, enhanced DNA repair, altered apoptotic signaling, immune suppression, and selection of resistant cellular subpopulations. While these mechanisms remain highly relevant, they do not fully explain how tumor cells repeatedly survive in the face of chemotherapy, radiation-induced oxidative damage, and chronic immune stress. Increasing evidence suggests that therapeutic resistance also reflects a broader adaptive state in which cancer cells reconfigure metabolism, stress response pathways, and organelle homeostasis to preserve viability under treatment pressure (121–123). Within this adaptive framework, mitochondria occupy a central position. Because many anti-cancer therapies exert at least part of their effects through mitochondrial injury, oxidative stress, metabolic disruption, or activation of mitochondria-dependent apoptosis, the ability of tumor cells to maintain mitochondrial fitness can strongly influence treatment outcome. A cervical cancer cell that preserves mitochondrial respiration, stabilizes redox balance, removes damaged mitochondria, and prevents mtDNA- or ROS-driven catastrophic stress is far more likely to survive therapy than one that cannot (124, 125). Thus, therapeutic resistance in cervical cancer should increasingly be understood not only as a genomic or signaling problem, but also as a mitochondrial adaptation problem.

This perspective has important implications for both mechanistic interpretation and therapeutic design. It suggests that resistance is not simply the absence of drug sensitivity, but an actively maintained phenotype supported by bioenergetic flexibility, mitochondrial quality control, and stress buffering. As discussed below, several distinct mitochondrial processes-including respiratory remodeling, mitophagy, redox adaptation, and regulation of cell death thresholds-appear to contribute to this resistant state in cervical cancer.

### 5.2 Mitochondrial respiration and metabolic plasticity in cisplatin resistance

Cisplatin remains a foundational component of systemic therapy and concurrent chemoradiotherapy for cervical cancer, yet resistance to cisplatin is a major determinant of treatment failure. One emerging theme is that cisplatin-resistant cervical cancer cells are not simply cells with enhanced drug efflux or reduced DNA adduct formation, but cells that have undergone broader metabolic adaptation (126, 127). In particular, maintenance of mitochondrial respiration and metabolic plasticity appears to be a crucial element of resistance.

Mitochondrial respiration contributes to treatment tolerance in several ways. First, it supports ATP generation under conditions where therapy disrupts proliferative signaling and imposes energetic strain. Second, it sustains biosynthetic and redox-supporting pathways that help cells repair damage and recover from stress. Third, preservation of respiratory capacity can prevent catastrophic bioenergetic collapse, thereby keeping cells alive long enough to activate additional survival pathways. In this sense, therapy-resistant cells are often not metabolically dormant; rather, they are metabolically reprogrammed to remain functional under pressure. Recent evidence in cervical cancer supports this view by showing that regulatory molecules linked to cisplatin resistance can promote mitochondrial respiration and oxidative metabolism (128, 129). These findings suggest that resistant cells may retain or even enhance mitochondrial bioenergetic competence as a means of surviving genotoxic treatment. Importantly, this does not contradict the presence of elevated glycolysis. Instead, it reinforces the concept that resistant cervical cancer cells exploit metabolic flexibility, using both glycolytic and mitochondrial programs according to need.

This metabolic plasticity provides a survival advantage under therapy-induced stress. Cisplatin exposure can damage nuclear and mitochondrial components, alter redox state, and impair normal biosynthetic flux. Cells capable of adaptively reallocating metabolic resources and maintaining mitochondrial function are better positioned to buffer these disruptions. Therefore, mitochondrial respiration should be viewed not merely as a background metabolic process, but as a functional contributor to cisplatin resistance and a potential therapeutic vulnerability in cervical cancer.

### 5.3 Mitophagy as a buffering mechanism against therapy-induced mitochondrial damage

A major consequence of chemotherapy and radiotherapy is mitochondrial injury. These treatments can disrupt mitochondrial membranes, increase ROS production, impair electron transport, and destabilize mitochondrial DNA and membrane potential. If the resulting damage accumulates beyond a tolerable level, mitochondrial apoptosis and irreversible cell death may follow. However, cervical cancer cells can counteract this threat through activation of mitophagy, which functions as a protective buffering mechanism by selectively removing damaged mitochondria before they trigger lethal stress responses (130). This is particularly relevant in the setting of cisplatin treatment. Cisplatin-induced mitochondrial injury can be substantial, especially in cells already exposed to chronic oncogenic and oxidative stress (131). Under such conditions, mitophagy offers a means of survival by limiting the buildup of dysfunctional organelles and preserving a healthier mitochondrial pool. Rather than allowing damage to propagate through the mitochondrial network, tumor cells can selectively eliminate compromised mitochondria and maintain organelle function at a level compatible with survival. From the perspective of therapeutic resistance, this process has profound implications. Mitophagy effectively lowers the intracellular burden of treatment-induced stress, reduces mitochondrial ROS overload, and delays activation of mitochondria-dependent apoptosis (132). In doing so, it helps convert potentially lethal therapy into a manageable stressor. This means that even when anti-cancer treatment successfully injures mitochondria, tumor cells may still evade death if their quality-control systems remain sufficiently active.

Importantly, mitophagy does not act in isolation. It is closely linked to mitochondrial dynamics, because fragmented or dysfunctional mitochondria are often preferentially targeted for removal. It is also connected to redox homeostasis, since selective clearance of damaged organelles can prevent excessive ROS accumulation. In cervical cancer, this integration makes mitophagy a particularly powerful adaptive program. Therapeutically, it suggests that blocking mitophagy could sensitize tumor cells to cisplatin or radiation by preventing recovery from mitochondrial damage and forcing damaged organelles to accumulate beyond the survivable threshold.

### 5.4 Redox homeostasis and the suppression of oxidative cell death

Therapeutic resistance in cervical cancer is also shaped by the ability of tumor cells to maintain redox homeostasis under treatment-induced oxidative stress. Many anti-cancer interventions, including cisplatin and radiotherapy, exert part of their cytotoxic effect by increasing ROS production and overwhelming antioxidant defenses. Because mitochondria are major sources of ROS as well as primary targets of oxidative injury, mitochondrial redox regulation becomes a critical determinant of whether oxidative stress results in adaptive signaling or cell death (133, 134). Resistant cervical cancer cells are likely to preserve survival by reinforcing antioxidant buffering systems and restructuring mitochondrial metabolism in ways that reduce oxidative vulnerability. This includes maintaining glutathione-dependent defenses, preserving NADPH-generating pathways, modulating electron transport activity, and controlling mitochondrial substrate flow. Such adaptations help prevent oxidative stress from escalating into membrane damage, mitochondrial collapse, and activation of death pathways. This redox resilience may also intersect with resistance to ferroptosis, an iron-dependent form of oxidative cell death characterized by lipid peroxidation (135–137). Although ferroptosis is mechanistically distinct from classical mitochondrial apoptosis, mitochondrial metabolism and oxidative balance can influence ferroptotic susceptibility by regulating redox buffering, metabolite transport, and oxidative pressure. In cervical cancer, emerging evidence suggests that mitochondrial-associated factors can suppress ferroptosis and thereby support resistance to cisplatin (138). This expands the concept of mitochondrial therapeutic resistance beyond apoptosis alone and highlights the broader role of mitochondria in shaping cell death vulnerability.

From a clinical perspective, these findings are important because they indicate that treatment-resistant cells are often not simply “drug-insensitive” in a narrow sense; rather, they are highly effective at maintaining a survivable oxidative state. If this redox buffering network could be disrupted, resistant tumor cells might be pushed past their oxidative tolerance threshold and resensitized to therapy. Thus, redox homeostasis represents both a hallmark of mitochondrial adaptation and a promising target for overcoming resistance in cervical cancer.

### 5.5 Mitochondrial apoptosis threshold and treatment sensitivity

Many anti-cancer therapies ultimately depend on the activation of apoptosis, and mitochondria play a decisive role in determining whether this response occurs. The mitochondrial apoptotic pathway integrates signals from DNA damage, oxidative stress, metabolic dysfunction, and pro-survival signaling networks. A key determinant of treatment sensitivity is therefore the apoptotic threshold: the level of cumulative intracellular stress required to trigger mitochondrial outer membrane permeabilization, release of cytochrome c and other pro-apoptotic factors, and irreversible execution of cell death.

In cervical cancer, mitochondrial reprogramming can raise this apoptotic threshold and thereby reduce treatment sensitivity (139, 140). Cells with stabilized mitochondrial membrane potential, reinforced anti-apoptotic signaling, effective mitophagy, and strong redox buffering are less likely to undergo mitochondrial collapse in response to chemotherapy or radiation. Even when DNA damage is substantial, the absence of sufficient mitochondrial commitment to apoptosis may allow cells to survive, repair, or enter a transient stress-tolerant state (141–144). In this setting, mitochondria serve as gatekeepers that determine whether damage is translated into death or merely into adaptive stress signaling. This concept also helps explain why interventions targeting autophagy, mitophagy, or mitochondrial dynamics can alter chemosensitivity. By disturbing mitochondrial homeostasis, these interventions may lower the apoptotic threshold and make tumor cells more responsive to treatment-induced injury. Conversely, if mitochondrial homeostasis remains intact, tumor cells may continue to survive despite ongoing therapeutic exposure. Thus, treatment sensitivity is not determined solely by the extent of upstream damage, but by whether mitochondrial adaptation can contain that damage below the point of no return. Understanding the mitochondrial apoptotic threshold is particularly useful for conceptualizing resistance in cervical cancer because it integrates multiple pathways into one functional outcome. Respiratory remodeling, mitophagy, redox control, and structural stability all converge on the same question: can the mitochondria still sustain life under stress, or have they crossed into irreversible death signaling? In resistant tumors, the answer is often the former. Targeting this threshold directly or indirectly may therefore be an effective strategy for resensitizing cervical cancer cells to therapy.

### 5.6 Mitochondrial adaptation and resistance beyond chemotherapy

Although cisplatin resistance is the most extensively studied example, mitochondrial reprogramming likely contributes to resistance beyond chemotherapy alone. Radiotherapy, for instance, induces oxidative damage, DNA injury, and mitochondrial stress, all of which are influenced by the mitochondrial state of the cell (145–147). Tumor cells with efficient redox control, stable mitochondrial function, and enhanced quality control mechanisms are more likely to withstand radiation-induced stress and recover proliferative capacity after treatment. This is especially relevant in cervical cancer, where radiotherapy remains a major component of standard management.

**Figure 4:**
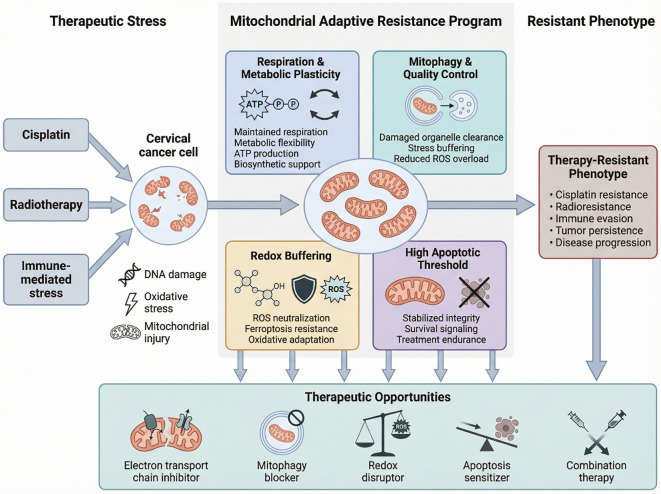
Mitochondrial reprogramming drives therapeutic resistance in cervical cancer. Under therapeutic stress, cervical cancer cells maintain survival through preserved mitochondrial respiration, adaptive mitophagy, reinforced redox buffering, and a high apoptotic threshold. Together, these interconnected mechanisms promote resistance to cisplatin, radiotherapy, and other stress-inducing treatments, while also revealing potential vulnerabilities that may be therapeutically targeted.

Similarly, mitochondrial adaptation may influence responsiveness to immune checkpoint inhibitors. In cervical cancer, where anti-PD-1-based regimens are now used in recurrent, metastatic, and high-risk locally advanced settings, tumor-cell mitochondrial fitness may affect whether immune-mediated pressure is translated into effective tumor killing. Enhanced OXPHOS, redox buffering, and mitophagy may help tumor cells tolerate T-cell-derived oxidative stress, reduce immunogenic mitochondrial danger signaling, and maintain survival despite checkpoint blockade. Conversely, therapeutic induction of mitochondrial stress may increase mtDNA-cGAS-STING activation, type I interferon signaling, and immunogenic cell death, thereby providing a rationale for combining mitochondrial perturbation with PD-1/PD-L1 blockade. If mitochondrial reprogramming supports immune evasion, dampens immunogenic stress signaling, or allows tumor cells to resist immune-mediated oxidative and apoptotic injury, then it may indirectly reduce the efficacy of checkpoint blockade or other immune-based approaches. In this framework, resistance to therapy is not divided neatly into separate categories of chemotherapy resistance, radio resistance, or immunotherapy resistance. Instead, these clinically distinct forms of failure may share a common biological denominator: mitochondrial fitness under stress. This shared denominator has important implications for combination treatment strategies. If mitochondria support survival across multiple therapeutic contexts, then mitochondrial-targeted interventions could potentially enhance the efficacy of several treatment modalities at once. For example, disrupting mitochondrial respiration, redox buffering, or mitophagy might not only increase cisplatin sensitivity but also amplify radiation damage or restore immune visibility. Such an approach would be especially valuable in cervical cancer, where multimodal therapy is common and resistance often emerges through overlapping rather than isolated mechanisms. Therefore, mitochondrial adaptation should be regarded as a broad resistance platform rather than a narrow mechanism tied to a single drug class. This broader perspective strengthens the rationale for therapeutic strategies aimed at weakening mitochondrial resilience in cervical cancer. As shown in **Figure 4**, therapeutic resistance in cervical cancer is supported by an integrated mitochondrial adaptive program that preserves bioenergetic function, buffers oxidative and structural damage, and prevents irreversible apoptosis. These interconnected mitochondrial mechanisms not only sustain treatment tolerance but also define potential targets for therapeutic resensitization.

### 5.7 Integrative view: mitochondrial fitness as a determinant of therapy response

Overall, mitochondrial fitness appears to be an important determinant of therapy response in cervical cancer. Respiratory remodeling, mitophagy, redox control, ferroptosis resistance, and regulation of mitochondrial apoptosis collectively allow tumor cells to tolerate chemotherapy-, radiotherapy-, and immune-mediated stress. Rather than representing isolated resistance mechanisms, these processes converge on a shared adaptive state in which cervical cancer cells preserve mitochondrial function under treatment pressure. **Table 1** summarizes representative studies linking mitochondrial mechanisms to therapeutic resistance in cervical cancer.

**Table 1:** Mitochondrial mechanisms underlying therapeutic resistance in cervical cancer.

Study	Main model/system	Resistance-related mitochondrial mechanism	Key findings	Relevance to this review
Liu et al.	Cervical cancer cells	Mitochondrial respiration	BRSK1 promoted cisplatin resistance by preserving mitochondrial respiration and metabolic adaptability.	Demonstrates that mitochondrial bioenergetic remodeling contributes directly to chemotherapy resistance.
Yang et al.	Cervical cancer cells	Mitochondrial transcription and OXPHOS	HMGCS1 mediated cisplatin resistance by regulating mitochondrial transcription and oxidative phosphorylation.	Supports the view that therapy-resistant cells maintain mitochondrial fitness under drug stress.
Chen et al.	HeLa cells	Mitophagy	Melatonin enhanced cisplatin-induced apoptosis by inhibiting the JNK/Parkin/mitophagy axis.	Indicates that mitophagy functions as a protective buffering mechanism against therapy-induced mitochondrial damage.
Mubthasima et al.	Cervical cancer models	Mitochondrial dynamics, autophagy, and mitophagy	Sesamol sensitized cervical cancer cells to cisplatin by modulating mitochondrial dynamics and suppressing adaptive autophagy/mitophagy.	Shows that mitochondrial structural adaptation and quality control are involved in treatment response.
Ma et al.	Cervical cancer cells	Redox regulation and ferroptosis suppression	SLC25A10 promoted cisplatin resistance by inhibiting ferroptosis and supporting oxidative stress tolerance.	Expands the resistance framework to include mitochondrial redox buffering and ferroptosis avoidance.
Sun et al.	CaSki cells	Mitochondrial apoptosis pathway	Beclin 1 influenced cisplatin-induced apoptosis through a mitochondria-dependent pathway.	Supports the idea that mitochondrial apoptotic threshold is a key determinant of treatment sensitivity.

## 6 Therapeutic opportunities targeting mitochondrial reprogramming

### 6.1 Why mitochondrial vulnerabilities are therapeutically attractive in cervical cancer

The recognition that mitochondrial reprogramming is central to cervical cancer progression, immune remodeling, and therapeutic resistance raises an important translational question: can mitochondria be exploited as therapeutic targets? This possibility is especially attractive because mitochondria are not peripheral contributors to tumor biology. Rather, they function as hubs that integrate energy production, biosynthesis, redox control, stress signaling, apoptotic commitment, and immune communication. Targeting such a central node offers the potential to disrupt multiple malignant phenotypes simultaneously rather than inhibiting a single downstream pathway.

It should be noted that the translational maturity of these strategies differs substantially. Some mitochondria-related interventions have been tested directly in cervical cancer cell or preclinical models, whereas others are supported mainly by evidence from other tumor types or by general principles of mitochondrial biology. Therefore, mitochondria-targeted therapeutic opportunities are discussed below according to their mechanistic plausibility and evidence strength, rather than as uniformly validated cervical cancer treatments.

In cervical cancer, this strategy may be particularly valuable for several reasons. First, the disease is driven by persistent HPV-associated oncogenic stress, which likely creates a chronic dependence on adaptive metabolic and mitochondrial programs. Second, standard therapies such as cisplatin and radiotherapy already place a strong burden on mitochondrial function, suggesting that tumor cells may exist close to a threshold of tolerable mitochondrial injury (148–150). Third, cervical cancer progression occurs within a distinct immunological context, meaning that mitochondrial-targeted interventions might reshape not only tumor cell survival but also immune responsiveness. These features make mitochondrial vulnerabilities especially relevant in both treatment-naive and resistant disease. From a broader therapeutic perspective, mitochondrial targeting is conceptually appealing because it can operate at several biological levels. Some interventions aim to impair mitochondrial metabolism directly, others destabilize mitochondrial dynamics or mitophagy, and still others seek to leverage mitochondrial stress to activate anti-tumor immunity. Rather than being a single treatment category, mitochondria-directed therapy should be viewed as a spectrum of strategies designed to interrupt tumor adaptation and expose stress-dependent vulnerabilities. At the same time, the therapeutic use of mitochondrial targeting requires careful conceptual framing. Because mitochondria are essential for normal tissue function and immune-cell fitness, the goal is not indiscriminate mitochondrial destruction. However, mitochondria-targeted therapy remains challenging because mitochondrial functions are also essential for normal epithelial cells, stromal cells, and anti-tumor immune cells. Therefore, therapeutic strategies must distinguish tumor-selective mitochondrial dependencies from mitochondrial processes required for normal tissue and immune-cell function. Instead, the challenge is to identify tumor-selective mitochondrial dependencies or context-specific windows in which cervical cancer cells are more reliant on mitochondrial adaptation than normal cells. The following sections summarize the major therapeutic avenues through which this goal may be pursued, with greater emphasis on representative agents, available clinical evidence, toxicity concerns, biomarker requirements, and practical barriers to clinical implementation. Importantly, the clinical translation of mitochondria-targeted therapy will require biomarkers capable of identifying tumors with specific mitochondrial dependencies, including OXPHOS addiction, enhanced mitophagy, redox adaptation, mtDNA-mediated immune signaling, or preserved immune competence.

### 6.2 Targeting mitochondrial metabolism and bioenergetics

One of the most direct therapeutic approaches is to disrupt mitochondrial metabolism and bioenergetic adaptation. Because cervical cancer cells often exhibit flexible use of glycolytic and mitochondrial pathways, blocking mitochondrial respiration or associated metabolic programs may limit the ability of tumor cells to survive under nutrient stress and treatment pressure (151). This strategy is especially relevant in resistant tumors, where preserved oxidative metabolism appears to support survival despite chemotherapy-induced damage. Targeting mitochondrial bioenergetics may produce anti-tumor effects through several mechanisms. Several representative agents illustrate the translational potential and limitations of targeting mitochondrial metabolism. Metformin, a commonly used antidiabetic drug, can inhibit mitochondrial complex I and reduce oxygen consumption, thereby potentially improving tumor oxygenation and radiosensitivity (152). In cervical cancer, metformin has been evaluated in clinical settings aimed at improving tumor hypoxia and response to chemoradiotherapy in locally advanced disease (153, 154). These studies support the feasibility of repurposing mitochondrial metabolism-modulating agents, although their efficacy as cervical cancer therapeutics remains to be fully established. Atovaquone, an antimalarial drug that inhibits mitochondrial complex III, has shown preclinical activity against cervical cancer cells by suppressing mitochondrial respiration and has been investigated clinically as a tumor hypoxia modifier in other solid tumors (155, 156). Devimistat (CPI-613), a lipoate analogue that targets mitochondrial tricarboxylic acid cycle enzymes, represents another mitochondria-directed therapeutic approach, although its clinical development has mainly focused on non-cervical solid tumors (157, 158). Therefore, these agents should be discussed as examples of mitochondria-modulating strategies with different degrees of cervical cancer-specific evidence rather than as validated treatments for cervical cancer.

Suppression of oxidative phosphorylation can reduce ATP availability, disrupt biosynthetic support, impair maintenance of membrane potential, and increase susceptibility to metabolic crisis (159–161). In parallel, interference with mitochondrial respiration may alter ROS production in ways that exceed the antioxidant buffering capacity of tumor cells, thereby pushing them toward oxidative damage and death. Importantly, these effects may be amplified in tumors already under therapeutic stress, such as those exposed to cisplatin or radiotherapy. This approach also aligns well with the concept of metabolic plasticity in cervical cancer. Resistant cells often survive because they can switch between metabolic states and preserve mitochondrial function when one pathway is challenged. Inhibiting mitochondrial metabolism may collapse this flexibility and narrow the adaptive range available to tumor cells. In this sense, targeting bioenergetics is not only about depriving cells of energy; it is about removing the metabolic resilience that underlies long-term survival. Nevertheless, metabolic targeting in cervical cancer is likely to be most effective when used in combination rather than as monotherapy. Because tumor cells can compensate through parallel pathways, strategies that inhibit mitochondrial metabolism may need to be paired with chemotherapy, radiotherapy, or inhibitors of redox buffering and autophagy. Such combinations may convert mitochondrial dependence from a background feature of cervical cancer biology into an actionable liability.

### 6.3 Targeting mitochondrial dynamics and structural adaptation

Mitochondrial dynamics represents another promising therapeutic entry point. Because the balance between fusion and fission is essential for maintaining mitochondrial network integrity, distributing metabolites, adapting to stress, and coordinating organelle quality control, disrupting this balance may impair tumor cell fitness. In cervical cancer, where mitochondrial network remodeling appears to support progression and survival, interventions that interfere with structural plasticity could destabilize the adaptive state of tumor cells.

Targeting mitochondrial dynamics may be therapeutically effective for several reasons. First, it can impair the ability of tumor cells to reorganize mitochondria in response to changing metabolic demand or therapeutic injury. Second, it may increase the accumulation of dysfunctional organelles by interfering with the coordination between mitochondrial fission and selective clearance. Third, it can alter apoptotic sensitivity, since mitochondrial morphology is closely linked to membrane integrity, cytochrome c release, and stress-induced death signaling (162–165). Together, these effects may weaken both basal tumor growth and resistance mechanisms. This strategy is conceptually attractive because mitochondrial dynamics lies at the crossroads of metabolism, mitophagy, and cell death. It is therefore less a purely structural target than a systems-level regulator of organelle fitness. In cervical cancer, where tumor cells must repeatedly adapt to oxidative, immunological, and treatment-related stress, compromising mitochondrial structural flexibility may have consequences far beyond morphology alone.

At present, the translational development of mitochondrial dynamics-targeted therapies remains relatively early, but the biological rationale is strong. Future progress will likely depend on identifying which components of the fusion-fission machinery are most selectively required in cervical cancer and how modulation of these pathways affects both tumor cells and infiltrating immune cells. If these questions can be resolved, mitochondrial dynamics may become a valuable therapeutic layer within broader mitochondria-based treatment strategies.

### 6.4 Blocking mitophagy to prevent recovery from mitochondrial injury

Because mitophagy functions as a central survival program in stress-exposed cervical cancer cells, its inhibition represents a compelling strategy for therapeutic sensitization. The rationale is straightforward: many treatments already damage mitochondria, but tumor cells often escape death by selectively removing the most dysfunctional organelles. If mitophagy is blocked, therapy-induced mitochondrial damage may accumulate to levels incompatible with survival, resulting in enhanced apoptosis, oxidative overload, or immunogenic stress signaling. This approach is particularly relevant for platinum-based therapy. Cisplatin can induce significant mitochondrial dysfunction, yet tumor cells with effective mitophagy may buffer this damage and maintain viability. Inhibiting mitophagy could therefore convert sublethal mitochondrial injury into irreversible organelle collapse (166). Such a strategy would not necessarily require generating new forms of stress; rather, it would prevent tumor cells from recovering from the stress already imposed by standard treatment. Blocking mitophagy may also have broader biological benefits. In addition to enhancing chemosensitivity, it could increase mitochondrial ROS accumulation, amplify release of mtDNA or other danger signals, and reduce the ability of tumor cells to maintain redox equilibrium. In this way, mitophagy inhibition may simultaneously impair tumor survival and increase immune visibility. This dual effect makes it especially appealing for cervical cancer, where mitochondrial stress and immune responsiveness are mechanistically intertwined.

However, as with all mitochondrial-targeted approaches, mitophagy inhibition must be considered carefully. Basal mitophagy is important for normal tissue homeostasis, and excessive blockade could affect nonmalignant cells or immune compartments. The challenge will therefore be to identify tumor-selective combinations or treatment windows in which cervical cancer cells are unusually dependent on stress-induced mitophagy. If successful, this strategy could become a powerful means of overcoming mitochondrial buffering and restoring therapy sensitivity.

### 6.5 Disrupting redox buffering and oxidative stress tolerance

Another promising strategy is to weaken the antioxidant systems that allow cervical cancer cells to survive persistent mitochondrial stress. Tumor cells often occupy a narrow redox window in which ROS levels are high enough to support signaling and adaptation but not so high as to trigger death. This balanced state is maintained through glutathione metabolism, NADPH-dependent buffering, regulation of mitochondrial substrate flow, and coordinated antioxidant networks. Disrupting these systems may push tumor cells beyond their oxidative tolerance threshold. This concept is highly relevant to therapeutic resistance because many anti-cancer treatments rely, at least in part, on oxidative damage. If tumor cells can efficiently neutralize ROS and repair oxidized macromolecules, therapy-induced stress may remain sublethal (167). In contrast, if redox buffering is impaired, the same therapeutic insult may become overwhelming. Thus, targeting oxidative stress tolerance can act as a sensitization strategy rather than a standalone cytotoxic mechanism.

In cervical cancer, disrupting redox homeostasis may also increase susceptibility to ferroptosis and other oxidative modes of cell death. This is particularly important because resistant tumors may suppress ferroptotic vulnerability through mitochondrial-associated metabolic adaptation. By weakening glutathione-based or metabolite-supported antioxidant defenses, it may be possible to expose a latent death pathway that standard therapies fail to engage fully. From a translational standpoint, redox targeting is appealing because it complements other mitochondria-based strategies. Inhibiting respiration can alter ROS production; blocking mitophagy can increase oxidative burden; and reducing antioxidant defenses can prevent recovery from either insult. Therefore, redox disruption may be most effective as part of rational combinatorial regimens that collectively erode mitochondrial stress tolerance in cervical cancer cells.

### 6.6 Leveraging mitochondrial stress to activate anti-tumor immunity

A particularly innovative therapeutic concept is to use mitochondrial stress not only to damage tumor cells directly but also to activate anti-tumor immunity. As discussed earlier, mitochondrial injury can result in release of mtDNA and other danger-associated signals that engage innate immune sensing pathways such as cGAS-STING (168). If appropriately triggered, this process may increase inflammatory visibility, enhance immune priming, and improve the responsiveness of cervical tumors to immunotherapy or conventional treatment. This approach is especially attractive in cervical cancer because the disease arises in the context of persistent viral oncogenesis and therefore has an inherent immunological dimension. In theory, tumor cells already contain viral antigens that could be recognized by the immune system. The problem is not necessarily absence of antigenicity, but failure of effective immune activation and persistence of an immunosuppressive microenvironment. Therapeutically induced mitochondrial stress may help bridge this gap by converting otherwise silent or buffered tumor-cell damage into signals that promote immune engagement.

The translational potential of this strategy lies in its dual action. On one level, mitochondrial stress can weaken tumor-cell survival; on another, it can increase the immunogenic consequences of that stress. This may create opportunities for synergy with checkpoint blockade, radiotherapy, or immunostimulatory agents. Rather than treating metabolism and immunity as separate domains, such approaches exploit the fact that mitochondrial signaling sits at their interface. At the same time, the success of this strategy depends on precise control. Excessive mitochondrial damage could harm beneficial immune cells, while chronic low-level activation might generate nonproductive inflammation without effective tumor clearance. The future of this field will likely depend on identifying dosing and combination strategies that favor acute immunogenic reprogramming rather than diffuse tissue stress. If those parameters can be optimized, mitochondria-directed immune activation may become one of the most distinctive therapeutic opportunities in cervical cancer.

### 6.7 HPV-directed and mitochondria-directed combination strategies

Given the central role of HPV oncogenic signaling in initiating the mitochondrial adaptive state of cervical cancer, a particularly compelling future direction is to combine HPV-directed interventions with mitochondria-targeted therapy. This approach is conceptually appealing because it addresses both the upstream driver and the downstream execution platform of malignant adaptation. Rather than targeting viral oncogenesis and mitochondrial reprogramming separately, a combined strategy may produce a more profound collapse of tumor fitness.

For example, therapies that reduce E6/E7 oncogenic activity may impair the proliferative and metabolic pressures that sustain cervical cancer cells, while mitochondria-targeted approaches could simultaneously remove the adaptive systems that allow those cells to survive under stress (169). This could be especially effective in resistant tumors, where HPV-driven transcriptional programs and mitochondrial stress-buffering mechanisms may cooperate to preserve viability. Combined targeting may therefore reduce the likelihood of metabolic escape and increase susceptibility to chemotherapy, radiotherapy, or immunotherapy. Such strategies may also improve specificity. HPV-directed interventions provide a disease-relevant layer of selectivity, whereas mitochondrial targeting attacks a central functional dependency of malignant cells. In theory, this pairing could produce a higher therapeutic index than broadly applied mitochondrial inhibitors alone. Moreover, because HPV-associated cervical cancer remains biologically distinct from many non-viral tumors, this dual-targeting framework may help define a more tailored therapeutic model for the disease.

Although still largely conceptual at present, the integration of HPV- and mitochondria-directed therapy represents one of the most promising translational directions emerging from current mechanistic research. It reflects the central thesis of this review: that HPV signaling, mitochondrial reprogramming, tumor immunity, and therapeutic resistance are not separate topics, but interconnected dimensions of cervical cancer biology that may need to be targeted together.

### 6.8 Representative agents, clinical trials, and translational barriers

Although mitochondria-targeted therapy is conceptually attractive, its clinical translation in cervical cancer remains at an early stage (170, 171). Several agents provide useful examples. Metformin is the most clinically accessible mitochondrial metabolism-modulating drug and has been investigated as a hypoxia-modifying agent in locally advanced cervical cancer, particularly in combination with chemoradiotherapy. Its rationale is based on inhibition of mitochondrial complex I, reduction of tumor oxygen consumption, and potential improvement of radiation response (172). However, the magnitude of mitochondrial inhibition achieved with clinically tolerable metformin doses may be modest, and patient selection based on tumor hypoxia or OXPHOS dependency is likely to be necessary.

Atovaquone is another repurposed agent with mitochondrial relevance. By inhibiting mitochondrial complex III, atovaquone can reduce oxygen consumption and tumor hypoxia. Preclinical studies suggest that atovaquone can suppress mitochondrial respiration in cervical cancer cells, but clinical studies of atovaquone as a hypoxia modifier have mainly been performed or initiated in other tumor types rather than cervical cancer (173–175). Therefore, atovaquone should currently be considered a promising extrapolated strategy that requires cervical cancer-specific validation. Other mitochondria-directed agents remain even more exploratory in cervical cancer. Devimistat (CPI-613), which targets mitochondrial tricarboxylic acid cycle metabolism, has entered clinical testing in advanced solid tumors but has not yet established a disease-specific role in cervical cancer (157, 158, 176). Autophagy or mitophagy-modulating agents, such as chloroquine or hydroxychloroquine, may theoretically sensitize tumor cells to chemotherapy or radiotherapy by preventing mitochondrial damage clearance, but these agents are not selective for mitophagy and may affect lysosomal function, immune-cell activity, and normal tissue homeostasis. Similarly, redox-modulating agents, ferroptosis-inducing strategies, mitochondrial antioxidants, and cGAS-STING-related approaches remain largely preclinical or mechanistically inferred in cervical cancer.

Several barriers must therefore be addressed before mitochondria-targeted therapies can be implemented clinically. First, mitochondrial functions are essential for normal tissues, stromal cells, and anti-tumor immune cells, raising concerns about systemic toxicity and immune impairment. Second, cervical cancers are metabolically heterogeneous, and only a subset of tumors may be truly dependent on OXPHOS, mitophagy, redox buffering, or mitochondrial stress signaling. Third, optimal combination partners, dosing schedules, and treatment timing remain undefined. Fourth, most current evidence is derived from cell-line models, whereas clinical translation will require HPV-defined models, patient-derived organoids, immune-competent systems, pharmacodynamic biomarkers, and prospective treatment-response cohorts. Thus, the therapeutic promise of mitochondrial targeting in cervical cancer should be viewed as biologically compelling but clinically immature.

### 6.9 Challenges and translational considerations

Beyond the selection of candidate agents, several cross-cutting challenges must be addressed before mitochondria-targeted strategies can be translated effectively into cervical cancer treatment. The first is selectivity. Because mitochondria are essential for the function of normal tissues and immune cells, systemic mitochondrial inhibition carries an inherent risk of toxicity. The goal is therefore not indiscriminate mitochondrial damage, but identification of context-specific dependencies that are stronger in cervical cancer cells than in normal cells. A second challenge is biological heterogeneity. Not all cervical tumors are likely to rely on mitochondrial reprogramming to the same degree. Some may be more dependent on oxidative metabolism, others on mitophagy or redox buffering, and still others on immune-modulatory mitochondrial signaling. This suggests that mitochondria-targeted therapies will likely require biomarker-guided patient stratification rather than uniform application. Identifying such biomarkers-such as signatures of OXPHOS dependency, mitophagy activation, redox adaptation, or STING competence-will be essential for rational clinical development. A third challenge is the dual role of mitochondria in both tumor cells and immune cells. Interventions designed to weaken tumor mitochondrial fitness could, if poorly calibrated, also impair T-cell function or innate immune responsiveness. This is especially important in the era of immunotherapy, where preserving or enhancing immune-cell competence is essential. Future therapeutic designs must therefore consider the tumor ecosystem as a whole rather than focusing exclusively on malignant cells. Finally, there is a need for better preclinical models that capture the full HPV-mitochondria-immunity axis. Traditional cancer cell lines may be useful for mechanistic studies, but they cannot fully represent the immunological and microenvironmental complexity of cervical cancer. More informative systems will likely include organoids, co-culture models, spatially resolved tumor profiling, and immune-competent *in vivo* models. These will be critical for determining which mitochondria-targeted strategies are most likely to succeed clinically.

### 6.10 Integrative view: targeting mitochondrial adaptation as a multi-level therapeutic strategy

Taken together, therapeutic targeting of mitochondrial reprogramming offers a multi-level strategy for cervical cancer treatment. It has the potential to impair tumor metabolism, destabilize organelle structure, prevent recovery from mitochondrial damage, disrupt redox buffering, enhance immunogenic stress signaling, and complement HPV-directed interventions. Few other therapeutic concepts provide such broad mechanistic reach. What makes mitochondrial targeting particularly compelling is that it addresses the same adaptive systems that underlie tumor progression, immune escape, and therapy resistance. In other words, it does not merely attack a downstream symptom of malignancy; it attacks the infrastructure that supports malignant persistence. This makes mitochondria not only biologically central but therapeutically strategic. At the same time, the success of this approach will depend on precision. Future progress will require identifying which mitochondrial vulnerabilities are most relevant in which patients, how they should be targeted in combination with existing therapies, and how to preserve beneficial immune function while weakening tumor resilience. With these considerations in mind, mitochondrial reprogramming may emerge as one of the most actionable integrative targets in cervical cancer. **Table 2** outlines emerging therapeutic strategies targeting mitochondrial vulnerabilities in cervical cancer and summarizes their biological rationale, translational promise, and current challenges.

**Table 2:** Emerging mitochondria-targeted therapeutic strategies and translational opportunities in cervical cancer.

Therapeutic strategy	Biological rationale	Representative evidence	Potential clinical implication	Key challenge
Targeting mitochondrial metabolism and oxidative phosphorylation	Cervical cancer cells rely on metabolic plasticity and preserved mitochondrial respiration for survival under stress.	Studies on BRSK1- and HMGCS1-associated cisplatin resistance support the importance of mitochondrial bioenergetics.	May enhance sensitivity to cisplatin and other stress-inducing therapies.	Limited tumor selectivity and possible toxicity to normal tissues.
Inhibiting mitophagy or mitochondrial quality control	Tumor cells use mitophagy to remove damaged mitochondria and buffer therapy-induced stress.	Parkin/mitophagy-related studies and autophagy-modulating sensitization strategies support this concept.	May convert sublethal mitochondrial damage into irreversible tumor-cell death.	Basal mitophagy is also important for normal-cell homeostasis.
Targeting mitochondrial dynamics	Fusion-fission balance supports tumor growth, stress adaptation, and apoptotic resistance.	Studies on Sp1-mediated mitochondrial network remodeling and Drp1-associated mitochondrial fission indicate therapeutic potential.	May destabilize mitochondrial fitness and reduce adaptive survival capacity.	The therapeutic window remains incompletely defined.
Disrupting redox buffering and ferroptosis resistance	Resistant tumor cells maintain survival by preventing oxidative stress from crossing lethal thresholds.	SLC25A10-associated suppression of ferroptosis supports redox-based resistance mechanisms.	May sensitize tumors to chemotherapy and oxidative damage.	Redox-targeting approaches may affect both tumor and immune cells.
Leveraging mtDNA-cGAS-STING signaling	Mitochondrial stress can activate innate immune pathways and increase tumor immunogenicity.	DMF-induced mtDNA-cGAS-STING activation in cervical cancer models demonstrates immunostimulatory potential.	May enhance anti-tumor immunity and improve response to immunotherapy or combination treatment.	Excessive or chronic activation may produce nonproductive inflammation.
Combining HPV-directed and mitochondria-directed therapies	HPV oncogenic signaling drives the adaptive mitochondrial state of cervical cancer cells.	Mitochondria-targeting compounds that also degrade HPV E6/E7 suggest dual-target potential.	May simultaneously suppress viral oncogenic dependency and mitochondrial adaptation.	Requires better biomarker-guided stratification and translational validation.

### 6.11 Biomarker-guided patient selection for mitochondria-targeted strategies

A clinically important question is how to identify cervical cancer patients who are most likely to benefit from mitochondria-targeted therapeutic strategies. Because mitochondrial reprogramming is heterogeneous, biomarker-guided patient selection will be essential for translating mitochondrial vulnerabilities into clinical practice (177, 178). Several categories of biomarkers may be considered. First, OXPHOS dependency could be evaluated using expression signatures of electron transport chain components, mitochondrial respiratory complex genes, mitochondrial biogenesis regulators such as PGC-1α and TFAM, or functional indicators of oxygen consumption and mitochondrial membrane potential (116, 179). Tumors with high OXPHOS dependency may be more vulnerable to inhibitors of mitochondrial respiration or metabolic plasticity.

Second, mitophagy-related biomarkers may help identify tumors that rely on mitochondrial quality control for survival under therapeutic stress. Increased expression or activation of PINK1, Parkin, BNIP3, NIX, LC3-associated mitophagy markers, or mitophagy-related transcriptional programs may indicate adaptive clearance of damaged mitochondria and potential sensitivity to combined mitophagy inhibition and chemotherapy or radiotherapy (80, 180–182). Third, redox adaptation markers, including NRF2 pathway activation, glutathione metabolism enzymes, thioredoxin-related proteins, SOD2, GPX family members, and ferroptosis-related regulators, may help identify tumors with enhanced oxidative stress tolerance (183, 184). Such tumors may be candidates for strategies designed to disrupt antioxidant buffering or restore oxidative cell death.

Fourth, biomarkers of mitochondrial DNA signaling may be useful for predicting immune-modulatory responses. Cytosolic or circulating mtDNA, cGAS-STING pathway activation, type I interferon signatures, and interferon-stimulated gene expression may reflect the extent to which mitochondrial stress is coupled to innate immune activation (91, 185). Finally, immune competence should be considered when designing mitochondria-based combinations with immunotherapy. Tumors with preserved antigen presentation, CD8^+^ T-cell infiltration, dendritic-cell activity, and a non-exhausted immune contexture may be more likely to benefit from therapies that convert mitochondrial stress into anti-tumor immunity. In contrast, tumors with severe immune exclusion or dominant suppressive myeloid infiltration may require additional immune-modulating strategies.

At present, most of these biomarkers remain exploratory in cervical cancer and require validation in clinical cohorts, treatment-response datasets, and patient-derived models. Future studies should integrate transcriptomic, proteomic, metabolomic, spatial, and functional mitochondrial assays to establish robust biomarker panels. Such biomarker-driven approaches may help determine whether mitochondrial targeting should be combined with chemotherapy, radiotherapy, immune checkpoint blockade, ferroptosis induction, or cGAS-STING activation in specific patient subsets.

## 7 Challenges, knowledge gaps, and future perspectives

### 7.1 Critical appraisal of current evidence

Although accumulating studies support the involvement of mitochondrial reprogramming in cervical cancer progression, immune regulation, and therapeutic resistance, the current evidence remains uneven across different mechanistic areas (88, 91, 186). Evidence linking mitochondrial metabolism, oxidative stress, mitophagy, and cisplatin resistance to cervical cancer biology is relatively more direct, as several studies have used cervical cancer cell lines or cervical cancer-related models (74, 187). In contrast, the roles of mitochondrial regulation in tumor immunity, immunometabolic competition, immune-cell mitochondrial fitness, and mitochondria-targeted immune remodeling remain less firmly established in cervical cancer and are partly extrapolated from broader cancer immunology and mitochondrial biology literature.

Several unresolved issues should be emphasized. First, it remains unclear whether mitochondrial reprogramming is a driver of cervical cancer progression or an adaptive consequence of HPV-driven transformation, chronic inflammation, and therapeutic pressure. Second, mitochondrial stress may have opposing biological outcomes. Acute mitochondrial damage and mtDNA release may activate innate immune signaling and enhance anti-tumor immunity, whereas chronic mitochondrial adaptation, redox buffering, and mitophagy may reduce immunogenic stress and promote immune evasion. Third, cGAS-STING activation may be beneficial in some contexts but may also generate chronic inflammatory signaling that does not necessarily translate into effective tumor clearance. Fourth, most available studies rely on established cervical cancer cell lines, which may not fully reproduce HPV genotype diversity, stromal interactions, immune-cell complexity, or the spatial organization of human tumors. Therefore, the mechanisms discussed in this review should not be interpreted as uniformly validated pathways in cervical cancer. Instead, they represent an evidence-graded framework in which some mechanisms are supported by direct cervical cancer studies, whereas others remain preliminary, context-dependent, or hypothesis-generating. Future studies using patient-derived tissues, HPV-defined models, organoids, immune-competent systems, spatial multi-omics, and treatment-response cohorts will be essential to determine which mitochondrial mechanisms are clinically relevant and therapeutically actionable in cervical cancer.

### 7.2 Direct mechanistic evidence in cervical cancer remains incomplete

Although increasing evidence supports a meaningful role for mitochondrial reprogramming in cervical cancer, the current literature remains uneven in depth and scope. Compared with broader work in pan-cancer metabolism or tumor immunology, mechanistic studies specifically addressing the mitochondria-immunity interface in cervical cancer are still relatively limited. Much of the available evidence focuses on tumor-cell intrinsic processes such as glycolytic regulation, mitochondrial dynamics, oxidative stress, or cisplatin resistance, whereas the direct relationship between mitochondrial remodeling and immune microenvironmental reprogramming has not yet been comprehensively defined (188–191). This limitation is important because cervical cancer is not simply another solid tumor. As an HPV-driven malignancy, it arises within a distinctive biological context shaped by chronic viral antigen exposure, inflammation, and host immune interaction. Therefore, conclusions extrapolated from non-viral tumors may not fully capture the mechanisms operating in cervical cancer. For example, while mtDNA-mediated cGAS-STING activation has been implicated in anti-tumor immune signaling in cervical cancer models, it remains unclear how broadly this pathway operates across patient subsets, disease stages, and treatment conditions. Likewise, the degree to which mitochondrial fitness directly influences immune escape in cervical cancer remains to be established with greater precision.

Another gap lies in the limited integration of mitochondrial pathways into a unified disease model. Many studies examine isolated processes-such as HK2 upregulation, mitochondrial respiration, mitophagy, or ferroptosis-related resistance-without clarifying how these events interact across the course of tumor progression. Yet the biological impact of mitochondrial reprogramming likely depends on the coordination of these processes rather than any one alteration alone. A stronger mechanistic framework will require studies that move beyond single-pathway description and instead examine how multiple aspects of mitochondrial adaptation combine to shape malignant behavior. Thus, one of the most urgent needs in this field is the generation of cervical cancer-specific mechanistic evidence that connects HPV signaling, mitochondrial remodeling, immune regulation, and therapy response within a coherent experimental system. Without this integration, the field risks remaining descriptive rather than truly translational.

### 7.3 The field must move beyond cancer-cell-centric models

Most available studies on mitochondrial reprogramming in cervical cancer are centered primarily on tumor cells. This focus has yielded important insights into metabolism, apoptosis, mitophagy, and drug resistance, but it leaves an incomplete picture of how mitochondrial biology influences the broader tumor ecosystem. In reality, cervical cancer progression depends not only on the intrinsic properties of malignant cells but also on dynamic interactions among tumor cells, immune populations, stromal cells, endothelial compartments, and extracellular metabolic conditions (192–194). A full understanding of mitochondrial reprogramming will therefore require moving beyond cancer-cell-centric models. This shift is especially important in the context of tumor immunity. Cytotoxic T cells, regulatory T cells, macrophages, dendritic cells, and other immune populations all rely on mitochondrial fitness for their activation, persistence, and function. If tumor cells are metabolically adapted while immune cells become energetically compromised or chronically stressed, the result may be an immunologically populated but functionally ineffective microenvironment. Yet in cervical cancer, the mitochondrial states of these nonmalignant compartments remain poorly characterized. We still know relatively little about how HPV-driven tumor metabolism affects immune-cell mitochondrial health, how mitochondrial stress signals influence myeloid-cell polarization, or how immune-cell dysfunction feeds back into tumor mitochondrial adaptation. This issue also has direct therapeutic relevance. Interventions that weaken mitochondrial function in tumor cells may have very different consequences in infiltrating lymphocytes or antigen-presenting cells. A therapy that appears effective in isolated cancer-cell models may prove less useful, or even counterproductive, if it compromises the immune compartment that is needed for durable anti-tumor control. Conversely, some mitochondrial-targeted strategies may enhance immune activation if they selectively increase tumor stress signaling while preserving immune-cell competence.

Future work should therefore treat mitochondria not merely as tumor-cell organelles but as regulators of multicellular ecosystem behavior. In cervical cancer, this broader perspective may be particularly valuable because the disease is shaped by persistent viral antigenicity, chronic immune interaction, and therapy-induced remodeling. Understanding how mitochondria coordinate these cross-compartment dynamics will be essential for both mechanistic progress and therapeutic design.

### 7.4 Better models and more advanced technologies are needed

The current methodological toolkit for studying mitochondrial reprogramming in cervical cancer remains limited. Traditional two-dimensional cell lines have been useful for defining specific pathways, but they cannot fully recapitulate the complexity of HPV persistence, immune interaction, stromal influence, and spatial metabolic heterogeneity found in actual tumors. Similarly, standard xenograft models often lack intact immune systems and therefore provide only a partial view of the mitochondria-tumor immunity interface. As a result, many important questions remain difficult to answer using conventional experimental systems alone.

To move the field forward, more physiologically relevant models are needed. These may include patient-derived organoids, HPV-informed three-dimensional culture systems, co-culture models integrating tumor and immune cells, and immune-competent *in vivo* platforms that better preserve microenvironmental context. Such models would allow researchers to study not only how mitochondrial pathways influence tumor-cell survival, but also how they shape immune recognition, cytokine networks, myeloid remodeling, and treatment response in more realistic settings (195, 196). In parallel, emerging technologies are likely to be transformative. Single-cell RNA sequencing, single-cell ATAC-seq, spatial transcriptomics, metabolomics, and multiplex imaging can provide insight into how mitochondrial states vary across cellular compartments within the cervical tumor microenvironment. Functional approaches such as metabolic flux analysis, live-cell mitochondrial imaging, and mtDNA stress profiling could further clarify how mitochondrial dynamics change over time and under treatment pressure. Importantly, integrating these datasets may reveal mitochondrial phenotypes that cannot be recognized through bulk analysis alone. Such tools will be especially important for resolving intratumoral heterogeneity. Not all cervical cancer cells within a tumor are likely to share the same mitochondrial dependencies, and not all immune cells are likely to experience the same degree of metabolic exhaustion or stress. Advanced technologies offer the possibility of identifying distinct mitochondrial subpopulations, stress-adaptive states, and therapy-resistant niches. These insights could ultimately support more precise therapeutic targeting and biomarker development.

### 7.5 Biomarker-guided stratification will be essential

One major obstacle to clinical translation is the lack of robust biomarkers that identify which cervical tumors are most dependent on mitochondrial reprogramming. It is unlikely that all patients will benefit equally from mitochondria-targeted strategies. Some tumors may rely heavily on oxidative phosphorylation, others on mitophagy or redox buffering, and still others on immune-modulatory mitochondrial signaling such as mtDNA-cGAS-STING activation. Without meaningful stratification, therapeutic trials may underestimate efficacy by treating biologically heterogeneous populations as though they were uniform. This highlights the need for biomarker-guided approaches. Potential markers may include signatures of mitochondrial respiration, expression of mitophagy-related genes, redox adaptation profiles, mtDNA stress indicators, or markers of STING pathway competence (197). More integrated biomarkers could combine mitochondrial features with HPV-related transcriptional activity, immune infiltration patterns, and therapy-response phenotypes. Such composite models may prove more informative than any single molecular marker alone.

Biomarker development is also important for understanding timing and therapeutic context. A tumor that is not highly mitochondria-dependent at diagnosis may become so after chemotherapy or under immune pressure. Similarly, mitochondrial immune signaling may be dormant at baseline but inducible during treatment. Longitudinal assessment may therefore be necessary to capture dynamic mitochondrial vulnerabilities rather than relying only on static pretreatment measurements. Ultimately, biomarker-guided stratification will be central to making mitochondria-targeted therapy clinically useful in cervical cancer. It will help define which patients should receive such approaches, at what stage of disease, and in combination with which therapies. Without this level of precision, mitochondria-targeted strategies may remain mechanistically intriguing but clinically inconsistent.

### 7.6 The dual role of mitochondria creates both opportunity and complexity

A recurring theme throughout this review is that mitochondria have a dual role in cervical cancer. On the one hand, they support malignant progression by sustaining metabolism, buffering oxidative stress, enabling mitophagy, and helping tumor cells survive treatment. On the other hand, they can generate danger signals, activate innate immune pathways, and potentially increase tumor immunogenicity when stressed appropriately. This duality is intellectually exciting, but it also creates substantial complexity for both mechanistic interpretation and therapeutic translation. For example, enhancing mitochondrial stress may increase tumor-cell death or immune activation, but it could also select for more stress-tolerant clones if not combined with adequate cytotoxic or immunologic pressure. Likewise, suppressing mitochondrial function may weaken tumor cells but also inadvertently impair anti-tumor immune cells if the intervention is too broad. The same pathway may thus be beneficial or harmful depending on timing, intensity, cellular context, and combination strategy. This complexity should not be viewed as a reason to avoid mitochondrial targeting. Rather, it underscores the need for precise intervention design. The goal is not simply to increase or decrease mitochondrial function in a generic sense, but to manipulate specific mitochondrial dependencies in ways that collapse tumor adaptation while preserving or enhancing beneficial immunity. In cervical cancer, where viral oncogenesis and immune interactions are central to disease biology, this precision will be particularly important.

Future research should therefore focus not only on identifying whether a mitochondrial pathway is involved, but also on defining the context in which it becomes therapeutically actionable. Understanding this context dependence will be one of the key steps required to move the field from mechanistic promise to clinical impact.

### 7.7 Future perspectives: toward an integrated HPV-mitochondria-immunity model

Looking forward, the most productive direction for this field is likely to be the development of an integrated model in which HPV oncogenic signaling, mitochondrial reprogramming, tumor immunity, and therapeutic resistance are studied as interconnected processes rather than separate domains. Such a model would treat mitochondria as both downstream effectors of HPV-driven transformation and active regulators of immune and therapeutic phenotypes. This would offer a more complete explanation of cervical cancer progression than models focused solely on viral oncogenes, metabolic pathways, or immune checkpoints in isolation. Within this framework, several research priorities emerge. First, it will be important to identify the specific mitochondrial programs most consistently engaged by HPV-transformed cells, including those involved in respiration, dynamics, redox control, and mtDNA signaling. Second, these pathways must be mapped across tumor and immune compartments to determine how mitochondrial states differ among cancer cells, T cells, macrophages, dendritic cells, and stromal populations. Third, researchers will need to define how these mitochondrial features evolve during treatment, recurrence, and immune escape. Equally important is the development of rational combination strategies. The greatest clinical benefit may come not from mitochondria-targeted monotherapy, but from integrating mitochondrial intervention with cisplatin, radiotherapy, immunotherapy, or HPV-directed therapies. Such combinations could simultaneously weaken tumor resilience, enhance immunogenic stress signaling, and reduce the likelihood of adaptive escape. The conceptual foundation for this approach is already emerging; what is needed now is systematic translational refinement. In this sense, the field stands at an important transitional stage. The evidence is sufficient to establish mitochondrial reprogramming as a meaningful component of cervical cancer biology, but not yet sufficient to define a mature translational paradigm. The next phase will require deeper mechanistic integration, more sophisticated experimental models, and clinically oriented biomarker strategies. If these advances are achieved, mitochondrial biology may become one of the most informative and actionable frameworks for understanding and treating cervical cancer.

## 8 Conclusion

Cervical cancer is a biologically complex malignancy in which persistent HPV oncogenic signaling, metabolic adaptation, immune remodeling, and treatment resistance are tightly intertwined. Within this landscape, mitochondria have emerged as far more than bioenergetic organelles. They function as central regulators of tumor-cell fitness by coordinating energy production, redox homeostasis, mitochondrial dynamics, quality control, apoptotic susceptibility, and danger-associated signaling. Through these diverse roles, mitochondrial reprogramming influences not only malignant progression but also the ability of cervical cancer cells to survive immune pressure and therapeutic stress. A key message of this review is that mitochondrial reprogramming should not be regarded as a secondary or isolated feature of cervical cancer. Rather, it is an active platform through which HPV-driven oncogenic programs are translated into adaptive malignant behavior. HPV oncogenes help establish a metabolic and mitochondrial state that favors persistent growth, while downstream mitochondrial processes-including respiratory remodeling, mitophagy, ROS buffering, and mtDNA signaling-shape tumor immunity, immune evasion, and therapeutic resistance. In this sense, the HPV-mitochondria-immunity axis provides a more integrated framework for understanding cervical cancer than models centered on any one component alone. This perspective also has important translational implications. If mitochondrial adaptation is essential for tumor persistence, then disrupting mitochondrial fitness may offer a way to impair multiple malignant phenotypes simultaneously. Strategies targeting mitochondrial metabolism, structural plasticity, mitophagy, redox buffering, and immunogenic stress signaling may complement existing therapies and help overcome resistance. At the same time, the dual role of mitochondria in both tumor and immune cells means that future interventions must be designed with precision and guided by biologically informed stratification. Overall, mitochondrial reprogramming represents both a conceptual lens and a therapeutic opportunity in cervical cancer. Continued efforts to integrate HPV biology, mitochondrial adaptation, tumor immunology, and treatment response will be critical for defining clinically actionable vulnerabilities. By moving toward this integrated view, the field may identify new biomarkers, refine combination therapies, and open more effective treatment avenues for patients with recurrent, metastatic, or therapy-refractory cervical cancer.
